# Supplementary material for: Speos: an ensemble graph representation learning framework to predict core gene candidates for complex diseases
Source: Nat Commun. 2023 Nov 8;14:7206. doi: 10.1038/s41467-023-42975-z (PMC10632370; doi:10.1038/s41467-023-42975-z)
Supplement: Supplementary file 1 — Supplementary Information [file 41467_2023_42975_MOESM1_ESM.pdf]

SUPPLEMENTARY INFORMATION

Supplementary Notes

Supplementary Note 1: Biases and PPI network selection

To estimate how the data generation process of different networks may affect predictions, we compared the topological measures for positively labeled Mendelian and the remaining genes. In systematically acquired large-scale datasets networks such as HuRI[1] and BioPlex[2], Mendelian disorder genes have almost the same probability to be in the neighborhood of other Mendelian disorder genes as unlabeled genes (Supplementary Figure 1a). In contrast, in networks generated by agglomerating data from hypothesis-driven approaches the proportion of other Mendelian disorder genes in their neighborhood (homophily) increases. This drastic discrepancy reflects on one hand the well documented inspection bias of hypothesis-driven approaches[1,3,4], but may also be influenced by the incompleteness of high-throughput approaches (Supplementary Figure 1b). The degree distribution of Mendelian and unlabeled genes further confirms the substantial inspection bias in the small-scale literature. While there is no meaningful difference in the distribution and mean degree of both groups of genes in the tested unbiased networks, in networks based on the small-scale literature, Mendelian disorder genes show a substantial shift towards higher degrees than unlabeled genes, this reflecting the ‘interest’ of the community in specifically studying these genes (Supplementary Figure 1c). Since GNNs are influenced by the label distribution in the direct neighborhood[5], this effect of publication bias on the neighborhood label distribution might artificially increase performance on a holdout set while at the same time perpetuating existing biases if hypothesis-driven networks are used. Although IntAct[6] shows biased patterns towards a higher mean degree of Mendelian disorder genes, we include it in our analyses both as a reference to other studies and to account for interactions that are not covered by high-throughput approaches, but we monitor the effect of its biases on the resulting candidate genes (Supplementary Note 4). STRING[7] (cutoff 0.7), on the other hand, is excluded due to its drastic difference in degree distributions between Mendelian disorder genes and unlabeled genes.

Supplementary Note 2: Design and optimization of GNN

Our model consists of three parts, pre-message passing (pre-MP), message passing (MP) and post-message passing (post-MP) (Supplementary Figure 5). Pre- and post-MP are MLPs built from stacked fully connected layers interspersed with ELU nonlinearity, while the MP module is built from GNN layers, interspersed with ELU nonlinearity and instance normalization layers. The pre-MP module is built of one input layer which transforms the input space into the hidden dimension dim_{hid} of our model and *q* further layers which extract node-level features and feed it into the MP module. We use the same hidden dimension dim_{hid} across all hidden layers of the model. The MP module is built from *r* blocks each consisting of one GNN, one nonlinearity, and one normalization layer, which then feeds the latent features into the post-MP module. The post-MP module consists of s fully connected layers for node-level pattern recognition and two fully connected layers to transform it into the one-dimensional output space with an intermediate step of $\frac{dim_{hid}}{2}$ dimensions. We have searched hyperparameters *q* and *s* for all iterations from 1 to 6 without much difference in performance (data not shown). Therefore we settled at *q* = *s* = *2*, which leaves both modules ample room for feature recognition and keeps the number of model parameters small. When evaluating the impact of the dimensionality of the pre- and post-MP input/output vectors dim_{hid} at 10, 20, 30, 40, 50, 75, 100, 125 we observed no performance change exceeding one standard deviation of the AUROC; we fixed dim_{hid} = 50. Although hidden dimensions larger than 125 were included in the code, the runs went out of memory on a 40GB A100 GPU for the large, multi-network models and did not return results.

For the MP module, we tested 35 adjacency matrices and eleven GNN layers from the repertoire of PyTorch Geometric, of which eight are single-network GNNs, which only use a single adjacency matrix and which are evaluated first, and three are multi-network GNNs, that can use multiple adjacency matrices simultaneously. Setting the number of GNN layers to *r* = *0* collapses the model into an MLP with only pre-MP and post-MP and renders the influence of adjacency matrix and GNN layer to zero. Supplementary Figure 6 shows the influence of different single-network GNN layers and the hyperparameter r on the performance of the model using the adjacency matrices BioPlex 3.0 HEK293T, GRNdb Adipose Tissue and IntAct Direct Interaction. We evaluated the topology adaptive graph layer (TAG)[8], GraphSAGE[9], the graph transformer layer[10], Chebyshev graph convolution layer[11], the seminal graph convolution layer (GCN)[12], simplified graph convolution layer (sGCN)[13], the graph attention layer (GAT)[14] and the graph isomorphism layer (GIN)[15]. We conducted the hyperparameter optimization for gene labels of immune dysregulation (Supplementary Figure 6) and cardiovascular disease (now shown). For two adjacency matrices, most layers reduce the model performance compared to an MLP (*r* = *0*). Only with IntAct Direct Interaction (Supplementary Figure 6c), the GNN (*r* > *0*) outperforms the MLP. However, across all three adjacency matrices, the TAG layer usually outperforms all other layers. The best performing layers, especially according to Supplementary Figure 6b, c are TAG, GraphSAGE and the graph transformer layer. These layers either have mechanisms to block out the neighborhood and retain the self node's features during the

convolution (TAG and GraphSAGE) or have a non-linear neighborhood attention mechanism (transformer), allowing them to modulate the incoming message. Simpler GNN layers, however, fail to perform compared to the MLP, which hints towards the tendency of the full neighborhood information being harmful for prediction. From these data we settled for 2 TAG layers which generally achieve the best performance and do not deteriorate performance below the performance of an MLP. After fixing the MP module to 2 layers of TAG, we investigated the performance on all 35 adjacency matrices. In this analysis we also included three multi-network GNN layers that use all adjacency matrices which are typed and fed in the network simultaneously: RGCN[16], RGAT[17] and FiLM[18]. RGAT went out of memory for all runs on a 40GB A100 GPU and is not shown. As recent analyses[19] showed that allowing the information flow to also bypass the GNN can drastically improve the performance, we also evaluated a skip-connection from pre-MP to the output of MP and concatenation of pre-MP's and MP's output features prior to post-MP (Supplementary Figure 5c). From the single-adjacency runs, only Intact Direct Interaction and Intact Physical Association provide a benefit, likely reflecting the above discussed bias (Supplementary Figure 7a). Using all adjacency matrices simultaneously is only helpful if the FiLM layer is used. Furthermore, using a skip-connection or concatenation is not beneficial, possibly because the TAG layer already contains skip-connections and the FiLM layer is flexible enough to block and override unhelpful information. We therefore settle on using the FiLM layer with all adjacency matrices and the TAG layer with IntAct Direct Interaction without any further skip-connections or concatenations.

Based on the HPO we have settled on two combinations of GNN layer and network: 1. using $r = 2$ TAG layers on the IntAct Direct Interaction network and 2. using $r = 2$ FiLM layers on a merge of all networks. Due to the strict annotation criteria the IntAct Direct Interaction network is very sparse (Supplementary Figure 7b) and most nodes are isolated, whereas after merging all networks for the FiLM layer, only 33 nodes are not in the large connected component (Supplementary Figure 7c). The average shortest path length between positives and between all genes are much shorter in the combined network than in IntAct Direct Interaction. However, in both graphs positives and unlabeled nodes do not differ in their average shortest path lengths. Also, when combining the networks, the node degree distribution shifts away from a scale-free graph as low-degree nodes become less frequent than mid-degree nodes. Finally, positives in Intact Direct Interaction have a much higher frequency of other positives in their neighborhood than when all networks are combined.

Supplementary Note 3: Network features influencing model performance

Several observations indicate that, while the input features of every node and adjacent edges are important for its own prediction, the features of neighboring nodes may be irrelevant or even harmful for the machine learning task. In the initial evaluation of base classifiers (Fig. 1a), the best performing method for recovering held out positives is N2V+MLP. This method first uses a random-walk based neural network to project the graph topology into vector space without any node features. These vectors, which describe the position of nodes in the graph, are concatenated with the regular input features like GWAS summary statistics and gene expression for the node of interest and feed it into an MLP. Since the MLP part of N2V+MLP processes every gene in isolation, the neighboring node's features are not seen by the model. It only processes the input features of the node itself along with the topological information of its position in the graph, encoding its connections to other nodes. At the same time we established that this topological information is valuable as N2V+MLP outperforms an MLP that only uses the regular input features in the recovery of held out positives (Fig. 1a) and in the discovery of unlabeled positives (Fig. 3a). In contrast to N2V+MLP, GNNs process graphs by convoluting the features of adjacent nodes along the connecting edges using the message passing framework. Thus, the edges are used to determine "where" the convolution is applied, but the neighboring nodes' features define the content that is processed and propagated through the network.

We observed that GNNs are outperformed by N2V+MLP in recovering held out positives (Fig. 1a), but perform better or comparable to N2V+MLP in discovering unlabeled positives (Fig. 3a, 4a). We ascribe this observation to the effect of GNN layers to regularize the ML training by weighting adjacent node's features and acting as a low pass filter, and thus lowering the risk of overfitting to known positive examples. However, the performance of different GNN layers varies greatly, and in fact several decrease overall model performance (Supplementary Figure 6), whereas others achieve high prediction performance also for novel core genes (Fig. 3d). N2V+MLP works well for known positives but underperforms for unknowns (Fig. 3c). Since we are predominantly interested in the discovery of unlabeled positives, GNNs are the more promising choice. However, GCN[12] and RGCN[16] layers generally underperform (Fig. 1b). On the one hand GCN, which mainly exploits the features of all neighboring nodes, is outperformed by an MLP, and on the other hand N2V+MLP, which exploits edges but no neighboring node features outperforms the MLP. So, It appears that the node features of neighbors are not helpful, while the edges are *per se* informative. This explains that TAG[8] ameliorates the dip in performance caused by the GCN layer by incorporating skip-connections, which can bypass the message passing. In this setting, the TAG layer can be understood as a conditional GNN layer which, if the adjacent node's features are unhelpful, can revert to an MLP for any given node. This is also mirrored in the fact that TAG always performs at least as good as an MLP if the network topology is unhelpful (Fig. 1b). However, this mechanism neglects the information contained in the topology of the graph alone, i.e. in the edges themselves, which appear to be helpful as indicated by the N2V+MLP performance. Conceptually, the FiLM[18]

layer, does not only use the sender node's features for its convolution, but also the receiving nodes' features and the type of the connecting edge. When we examined which features are most important for FiLM predictions we found that the influence of the adjacent node's input features was negligible for individual examined nodes (see Supplementary Figure 17), and globally the latent features of surrounding nodes make up only a tiny proportion of all messages in the FiLM layer (Supplementary Figure 18, Supplementary Note 6). This indicates that also for FiLM the receiving node's features and the edge type are the most important factors determining the message (Supplementary Note 7). Thus, reminiscent of the N2V+MLP results, the FiLM layer predominantly learns the topology free of the influence of adjacent node's features but has the additional advantage of incorporating edge types.

The fact that FiLM's messages are mostly dependent on the receiving node's features and the edge type is also reflected in the results shown in Fig. 5. The importance of the edges is almost identical between edges of the same type, with only minute differences caused by the sending node's features. It also indicates that only a handful of roughly 300 incident edges is important for the prediction. Collectively these analyses indicate that methods gain performance when they have the option to ignore adjacent nodes' features and instead learn patterns of select incident edges. Biologically, it is possible that neighborhood functions are encoded in the network topology, e.g. in the form of protein complexes, or a sufficiently specific interaction wiring in different modules, and thus learned indirectly by the GNNs as a pattern that better captures functions than individual node features. In addition, the wealth of connections, and network incompleteness, likely also impact on the observed phenomena. Novel methods that are designed to distill the information of edges[20,21] or topological features[22,23] of the graph alongside the node features could therefore be a valuable addition to future iterations of comparable work.

Supplementary Note 4: Impact of network biases on predictions

As the bias of aggregating small-scale literature is visible in network characteristics concerning Mendelian disorder genes (Supplementary Note 1), we monitored how these biased inputs affect the predicted candidate genes and potentially inhibit new insights. TAG, which is trained only on IntAct Direct Interaction, is heavily biased towards predicting genes that have a degree larger than zero in the IntAct Direct Interaction network (Supplementary Figure 11a) and in the case of immune dysregulation even exclusively predict such genes as candidates (Supplementary Figure 11b). This indicates that presence in the IntAct direct network, and hence the fact that the involved proteins were deemed ‘interesting’ by researchers to justify their biochemical purification and *in vitro* interaction studies, and hence previous perceptions of a gene’s importance, was a key feature in their prediction by TAG. The argument that the scientific communities’ accumulated knowledge reflected in such a focused deeper characterization of relatively few genes corresponds to underlying biological importance has previously been refuted[1,3,4]. The candidate genes predicted by FiLM, which is trained on all networks simultaneously including IntAct Direct Interaction and is aware of edge types, shows much lower odds ratios (OR) for genes from IntAct Direct Interaction, but still significantly higher than 1 (Supplementary Figure 11a). The Node2Vec in N2V+MLP is also trained on all networks simultaneously but is not aware of edge types, so IntAct Direct Interaction makes up only 0.36% of all edges, thus further reducing the ORs of genes contained in IntAct Direct Interaction among its candidates. Importantly, when FiLM is trained on all networks except IntAct Direct Interaction and IntAct Physical Association (FiLM Unbiased), we still reap the benefits of GNNs without biasing predictions towards genes included in IntAct. However, even the candidates produced by N2V+MLP and FiLM Unbiased are weakly enriched for genes involved in IntAct Direct Interaction, reflecting a bias of IntAct Direct Interaction for immune system regulation. This interpretation is supported by the validation of FiLM Unbiased predictions using mouse KOs, in which the OR drops for immune dysregulation, compared to the initial FiLM predictions, but not for other disease groups like cardiovascular disease (Supplementary Figure 12a, Supplementary Data 4). Furthermore, differentially expressed genes and drug targets (Supplementary Figure 12b, c) show comparable levels of enrichment for most diseases whether IntAct Direct Interaction is used with FiLM or not. Intriguingly, for immune dysregulation the enrichment of differentially expressed genes increases when IntAct data are removed, indicating that these networks might also be biased towards genes examined in small-scale mouse experiments, which is consistent with the reasoning that laborious mouse knock-out and in vitro studies are more readily done for genes/proteins considered important. Moreover, for immune dysregulation only candidates produced by the unbiased version of FiLM are enriched for druggable genes which are not yet drug targets (Supplementary Figure 12c, Dr-), indicating that the biases inherited from small scale literature could indeed prevent the discovery of new drug development opportunities.

Supplementary Note 5: Extraction of sub-phenotypes

Upon closer examination, the query which leads to the Mendelian genes for cardiovascular disease mixes two distinct phenotypes: 1) a phenotype that is more focused on the heart muscle, its insufficiency or anatomical anomalies and 2) a phenotype that is mostly focused on the coronary arteries, stenosis and the cumulative deposits of plaque consecutively leading to disease. We therefore hypothesized that the unexpected results in the LoF validation are rooted in the classifier's inability to fully capture these two distinct phenotypes. To test this hypothesis in an ablation experiment, we have split the query terms into two lists, 'heart disease' and 'coronary artery disease', to more precisely capture the two subtypes. We used these queries to obtain new sets of Mendelian genes from OMIM, which we conditioned to be strict subsets of the Mendelian genes previously used for cardiovascular disease, to not dilute the meticulous pre-selection carried out by Freund *et al.*[24]. The query for heart disease returned 400 Mendelian disorder genes while the query for coronary artery disease returned 271 Mendelian disorder genes with an overlap between the two sets of size 134 (Supplementary Data 21). We used both lists to train new ensembles and repeated the LoF validation with the resulting candidate genes. Once the two phenotypes are separated, the LoF validation turns out more in line with our understanding of core genes (Supplementary Figure 15a & b), while the rest of validations still return strong results (Supplementary Figure 15c-e). We therefore conclude that our hypothesis is plausible; it appears that, in this instance, mixing of two distinct phenotypes caused the unexpected result. The newly introduced depletion in the results of the GCN classifier can be attributed to its generally mixed performance in several validations.

Supplementary Note 6: Importance of neighborhood node features

We have shown that some edges in the direct neighborhood of nodes and a selection of input features are vital for classification (Fig. 5). Besides their own input features, other node's input features appear to be irrelevant for the prediction (Supplementary Figure 17). Some of the genes (e.g. HBB) are not even in the 2-hop neighborhood of the query node, indicating that the threshold of 0.1 (dotted gray line) is a good threshold to separate signal from statistical noise. The fact that TNFRSF25 and TNFRSF6B's input features do not seem relevant, even though their connection to TNFSF15 is relevant (Fig. 5) indicates that it is in fact the physical connection of the proteins (i.e. the edge), and not the neighboring node's features, that is meaningful. It furthermore underlines the power of the FiLM layer, which can override the message coming from these nodes and thus can create a meaningful message from unhelpful incoming node features (see Methods Section for details). Since the overridden message is conditioned only on the receiving node and the connecting edge type, the message is identical for all incoming connections of the same type. This explains why the edge importance of edges of the same type are almost identical (Fig. 5) and why RGCN, which lacks this mechanism and relies on transformations of the sender's features, fails to perform adequately compared to FiLM (Fig. 1b).

To further investigate the conjecture that neighboring node's features are mostly irrelevant for the

prediction while the edges themselves are relevant, we have ascertained the influence of sender, receiver and edge on the message passing dynamic of the FiLM layer. The FiLM layer introduces an offset beta and a linear coefficient gamma for every feature of an incoming message $\mathbf{x}_u^{(t)}$ from the sender node u in the neighborhood of v based on the edge type r and the receiver node v:

$$\mathbf{x}_v^{(t+1)} = \sum_{r\in R} \sum_{u\in N(v)} \sigma(\boldsymbol{\gamma}_{r,v}^{(t)} \odot \mathbf{W}_r \mathbf{x}_u^{(t)} + \boldsymbol{\beta}_{r,v}^{(t)}) \quad (1)$$

Thus, the influence of the neighborhood node's features is only relevant for the first part of the term:

$$\boldsymbol{\gamma}_{r,v}^{(t)} \odot \mathbf{W}_r \mathbf{x}_u^{(t)}\ , (1)$$

while the bias $\boldsymbol{\beta}_{r,v}^{(t)}$ is only dependent on the receiver node v and the edge type r. We can therefore assess the balance between the influence of the neighborhood node's features and the features of the receiving node the following ratio:

$$\frac{\boldsymbol{\gamma}_{r,v}^{(t)} \odot \mathbf{W}_r \mathbf{x}_u^{(t)}}{\boldsymbol{\beta}_{r,v}^{(t)}}\ , (3)$$

which is close to 0 if the message is dominated by the bias term $\boldsymbol{\beta}_{r,v}^{(t)}$ and thus irrelevant of the neighborhood node's features. A high value still does not guarantee a high influence of $\mathbf{x}_u^{(t)}$, but we can assume that it is increasingly relevant if the model decides to modulate it via $\boldsymbol{\gamma}_{r,v}^{(t)}$ instead of simply overriding it via $\boldsymbol{\beta}_{r,v}^{(t)}$.

The message features passed along the edges of all networks of the first FiLM-layer are, in fact, dominated by the bias term $\boldsymbol{\beta}_{r,v}^{(t)}$, rendering the sender's latent features irrelevant (Supplementary Fig. 18a). The message features passed along the edges of gene regulatory layers by the second layer (see Supplementary Fig. 18b) are less heavily dominated by the bias term. However, this still does not mean that the actual input features are important, since the latent features of the senders have already been influenced by their incident edges in the first layer. It does, however, indicate that protein-protein networks and gene regulatory networks convey different notions of neighborhoods, which might be influenced by the former being bidirectional and the latter being unidirectional.

Supplementary Note 7: Additional predicted examples

Analogous to the immune dysregulation examples in the main text, we also explored candidate genes predicted by FiLM for cardiovascular disease for suitable targets for drug development. OBSCN and ITGA7 receive high Consensus Scores (11 and 9, respectively) and their protein products are druggable but not yet targeted by any drug.

OBSCN encodes the protein obscurin and is a large, modular protein with more than 80 exons and 28 transcript isoforms[25], which fulfill a wide range of functions in different tissues including skeletal and heart muscle[26]. Specific mutations in the OBSCN gene are implicated in hypertrophic cardiomyopathy[27], age-dependent cardiac remodeling and arrythmia[28]. Furthermore, obscurin has been implicated in non-muscular functions and pathologies[26]. Film bases its prediction of OBSCN as candidate gene on its location downstream of multiple transcription factors across several tissues, and implicating it in inflammation (STAT2, ZNF384)[29–31], angiogenesis (BRF1)[32], and immune dysregulation after ischemic damage (IRF3)[33] (Supplementary Figure 16a).

The integrin subunit alpha 7 encoded by ITGA7 is located in the cell membrane and involved in cell-cell and cell-matrix communication, and has been implicated in migration and invasion of malignant cells in metastasis formation[34,35]. Recently it was shown that mutations in ITGA7 contribute to congenital muscular dystrophy[36], adult-onset cardiac dysfunction[37] and cardiomyopathy[38,39], implicating a role in the etiology of cardiovascular diseases. The fact that FiLM bases its prediction on ITGA7 being located downstream of the estrogen related receptor alpha, encoded by ESRRA (Supplementary Figure 16b), opens the possibility that this gene mediates sex-related differences in genetic cardiomyopathies[40].

Neither OBSCN nor ITGA7 have been detected in GWAS studies for any heart-related traits, except PR interval (GCST010321) in the case of OBSCN. Despite this lack of detection, some OBSCN variants are known to contribute to left ventricular compaction[41] and dilated cardiomyopathy[42]. Despite the absence of GWAS signal, Speos identified them as core gene candidates based on their tissue-specific gene expression (Supplementary Figure 16c, d). Especially their high expression in the left ventricle and atrial appendage are vital for their classification as expected for factors contributing to cardiovascular disease, as these anatomical regions are key players in several related pathophysiologies[41,43]. Although the understanding of the role the two genes play in cardiovascular disease is still in its infancy, at least OBSCN already raised expectations for novel treatments and therapeutics[26], underscoring the value of Speos' predictions for hypothesis development, even when significant genome wide associations have not been detected.

Supplementary Figures

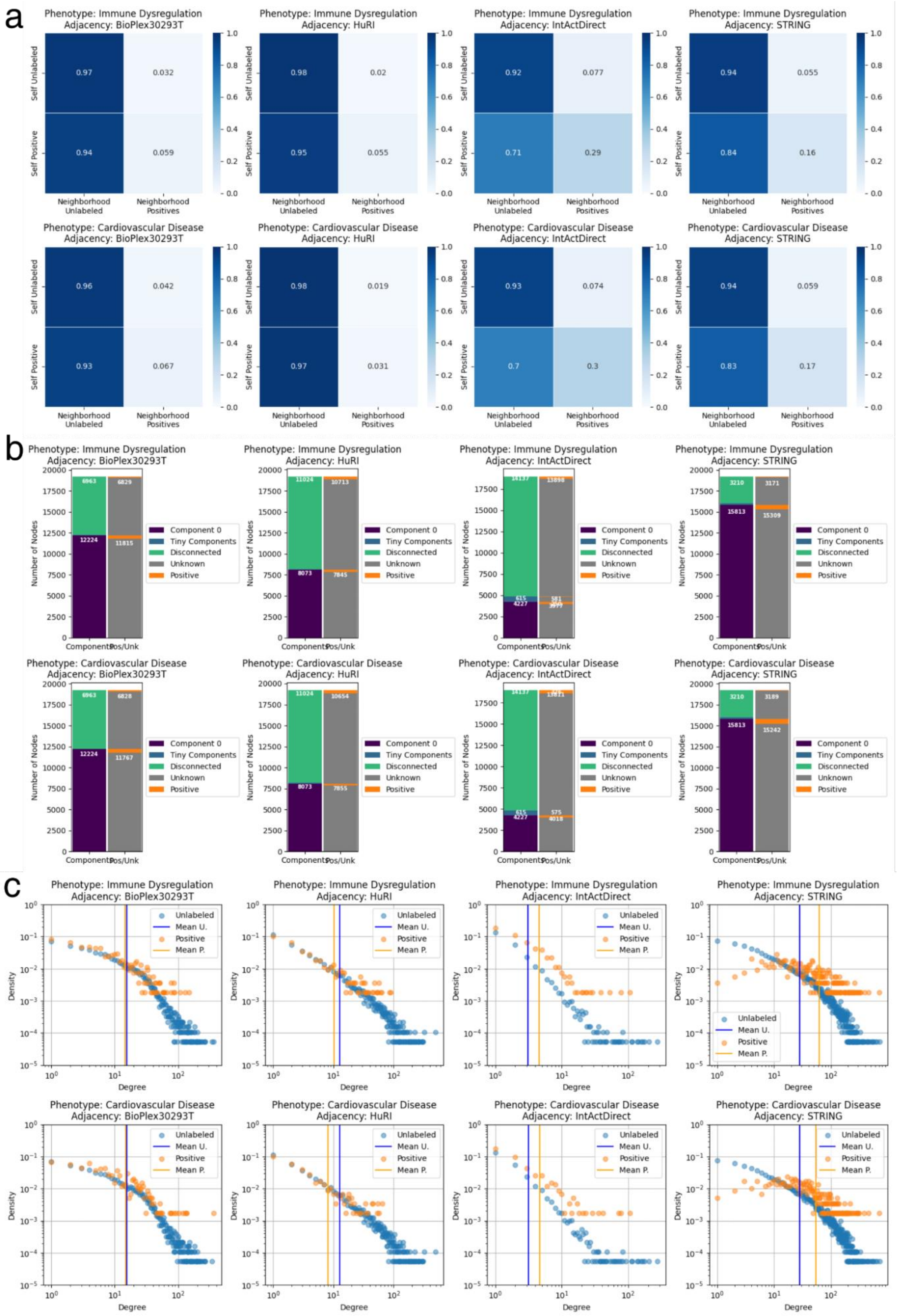

Supplementary Figure 1 | Properties of biased and unbiased networks. a, the top and bottom rows show the dependency of the neighborhood labels on the label of the center node of unlabeled genes

and Mendelian disorder genes for immune dysregulation and cardiovascular disease, respectively. The two left columns show results for systematically generated Bioplex 3.0 HEK293T and HuRI adjacency matrices. The two right columns show the adjacency matrices IntAct Direct Interaction and STRING (confidence > 0.7), which are largely assembled from hypothesis-driven small-scale data. Connectivity of Mendelian gene encoded proteins in the systematic networks is similar to that of unlabeled nodes. In the collated networks, proteins encoded by Mendelian disorder genes show higher assortativity, i.e. tendency to interact with each other, for both phenotypes. **b**, in each panel the left bar shows the fraction of nodes in the largest connected component (component 0) versus isolated small components and disconnected nodes. The right bar shows how the positive and unlabeled nodes are distributed among these components. **c**, the top and bottom rows show the degree distributions of Mendelian disorder genes and unlabeled genes for immune dysregulation and cardiovascular disease, respectively. The two left columns show the adjacencies Bioplex 3.0 293T and HuRI, which are unbiased, systematically generated networks. The two right columns show the adjacencies IntAct Direct and STRING (confidence > 0.7), which are not systematically generated. The bias towards known disease genes in the two right networks can be seen for both phenotypes. First, the average degree of Mendelian disorder genes is higher than the average degree of unlabeled genes. Second, the degree distribution of the Mendelian disorder genes in STRING does not follow a scale-free degree distribution. On the contrary, nodes with a medium degree are the most abundant, while nodes of low and very high degree are rare.

Method Reference	class prior assumption-free*	ensemble	unbiased networks**	networks extensible***	systematic external validation****	uses random walks	uses graph convolutions	uses input features	input extensible***
PRINCE[38]	✗	✗	✗	✗	✗	✗	✓†	✗	n.a.
Yang, 2014[39]	✗	✓	✗	✗	✗	✗	✓†	✓	✗
DWPC[32]	✗	✗	✗	✗	✗	✓	✗	✗	✓
Huang, 2018[33]	✗	✗	✓	✗	✓	✓	✗	✗	n.a.
RWR-MH[34]	✗	✗	✗	✗	✗	✓	✗	✗	n.a.
RWRHN-FF[35]	✗	✗	✗	✗	✗	✓	✗	✗	n.a.
EMOGI[40]	✗	✗	✗	✓	✓	✗	✓	✓	✓
KGED[41]	✗	✗	✗	✗	✗	✗	✓°	✓	✗
Du, 2021[42]	✗	✗	✗	✗	✗	✓	✗	✗	n.a.
CIPHER-SC[43]	✗	✗	✗	✗	✗	✗	✓	✓	✗
Graphene[44]	✗	✗	✗	✗	✓	✗	✓	✓	✗
Speos (Ours)	✓	✓	✓	✓	✓	✓	✓	✓	✓

Supplementary Figure 2 | Methods Comparison. *) assumptions about the prior class distribution of the PU learning problem in form of arbitrary cut-off that are imposed on rank distributions to divide candidate and non-candidate genes, in form of predetermined proportion of unlabeled positives. **) positive if the article also reports results using only unbiased networks or if the method can be reduced to only use unbiased networks without having to re-implement it. “Unbiased” networks represent large-scale, systematic experiments whereas “biased” networks stem from aggregating small-scale literature or using disease or gene ontologies. ***) within reason, i.e. without having to re-implement the method. ****) negative if the labels for validation are sourced from the same database as the input data, networks or training labels. †) uses label propagation instead of GNNs. °) Uses knowledge graph embedding models instead of GNNs.

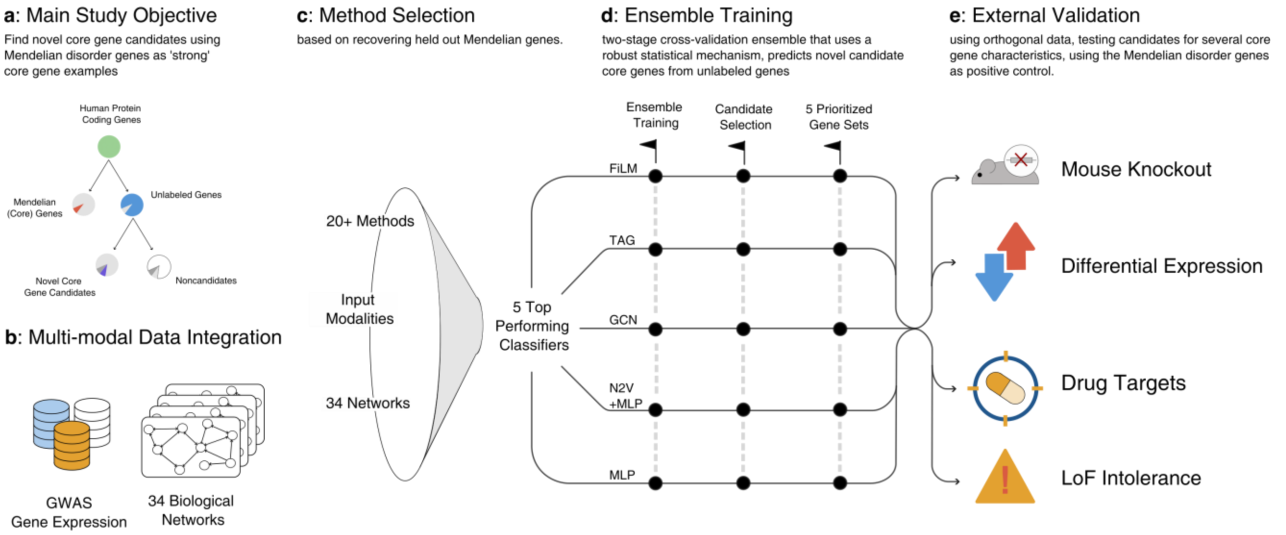

Supplementary Figure 3 | Graphical Overview. a: The main objective of the study is to predict novel core genes for common complex diseases using Mendelian disorder genes as ground truth “strong” core genes according to the omnigenic model. b: We integrate multi-modal data either as feature vectors or as network data. A feature vector containing GWAS and gene expression features is assigned to every gene, genes with missing data are discarded. Genes are connected by several different biological networks which can be undirected or directed. Each network can be used individually or in conjunction with other networks. c: Hyperparameter search testing for various variations of networks, input data and methods to identify the method and setting that is best suited to recover held out Mendelian disorder genes. d: Development of a nested crossvalidation ensemble that leverages consensus between models in a two-stage process to predict novel core gene candidates from unlabeled genes. The best performing classifiers from step c are used as base classifiers for the ensemble. e: The core gene candidates identified in step d are selected for external validation by testing for several criteria that are expected for core genes, such as disease-specificity in knockout experiments and differential expression when the disease is present.

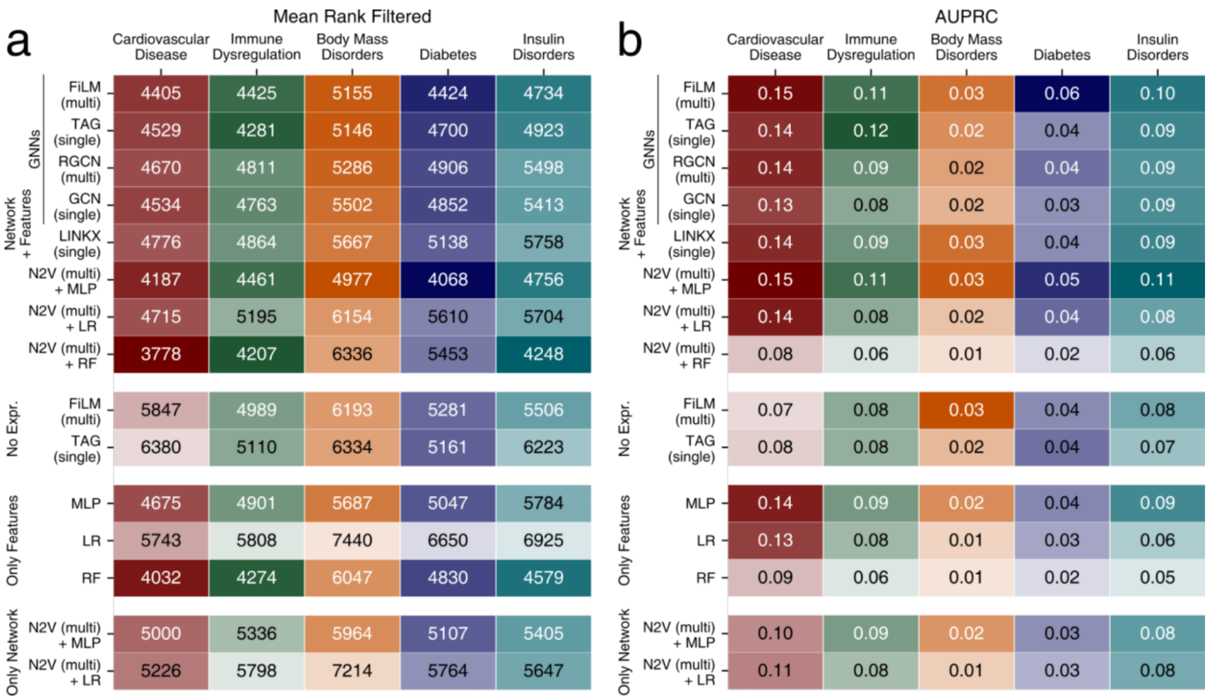

Supplementary Figure 4 | Additional performance metrics. a, the mean rank of all held out positives, ranked individually (i.e. filtered) against all unlabeled genes (lower is better), for different base classifiers, dataset variants and phenotypes. Combinations of methods and input data are indicated along the y-axis. The blocks group models using common input data as indicated: Only Network: adjacency matrix/matrices; Only Features: gene expression and GWAS input features but no adjacency matrices; No Expression: GWAS input features and adjacency of individual (single) or multiple (multi) networks; Network + Features: adjacency of individual (single) or multiple (multi) networks, GWAS and gene expression. b, the mean area under the precision recall curve (AUPRC) metric (higher is better) for different base classifiers, dataset variants and phenotypes. Combinations of methods and input data are indicated along the y-axis. The blocks group models using common input data as indicated: Only Network: adjacency matrix/matrices; Only Features: gene expression and GWAS input features but no adjacency matrices; No Expression: GWAS input features and adjacency of individual (single) or multiple (multi) networks; Network + Features: adjacency of individual (single) or multiple (multi) networks, GWAS and gene expression.

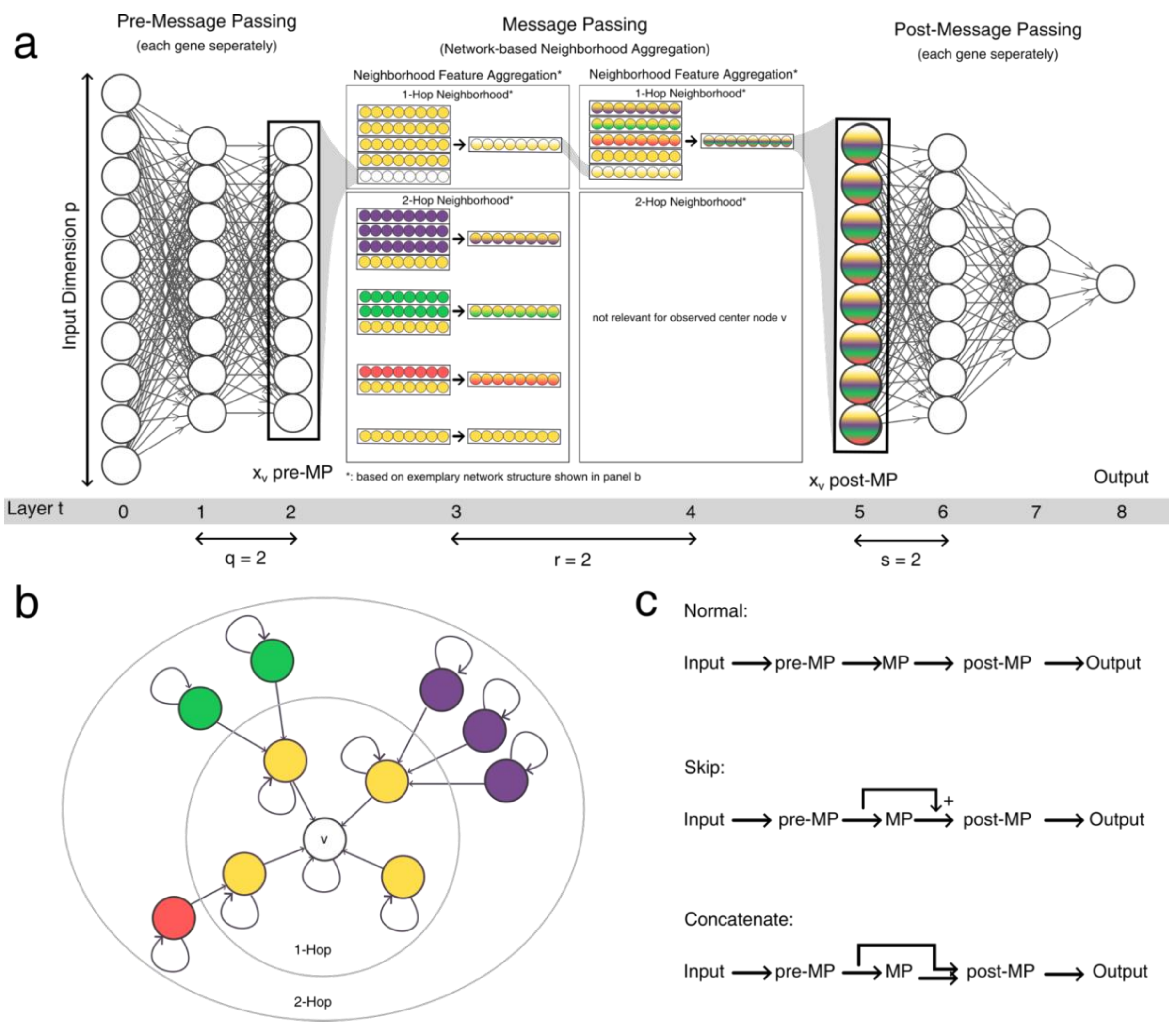

Supplementary Figure 5 | GNN Model Architecture. a, the general model architecture of all GNN models used in the experiments. The input features of node v are transformed into latent space by the pre-message passing module, which produces the latent vector $\mathbf{x}_v$ pre-MP. This latent vector is fed into the message-passing module, where the neighborhood feature aggregation takes place according to the graph shown in panel b. Each layer aggregates one hop in the network. Arrows denote the aggregation operators of the respective GNN layers described in the Methods section. After message passing, the latent vector $\mathbf{x}_v$ post-MP contains information of its n-hop neighborhood and is fed into the post-message passing module, which predicts the class of node v. The hyperparameters q, r and s control the number of layers per module. Not shown are nonlinearity functions and normalization layers. b, the simplified graph structure for the message passing shown in a with the observed node v in the center. Arrowheads denote the direction of the message passing; circles denote the respective n-Hop neighborhoods. c, normal versus alternative information flow through the network. Most commonly, all modules are chained consecutively, each feeding its output to the next. In the 'Skip' setting, the output vectors of the pre-MP and of the MP are summed up before being fed into the post-MP module. In the 'concatenate' setting, the output vectors of the pre-MP and of the MP are concatenated before being jointly fed into the post-message passing module. In this setting, the first layer of the post-message passing module has twice the number of dimensions.

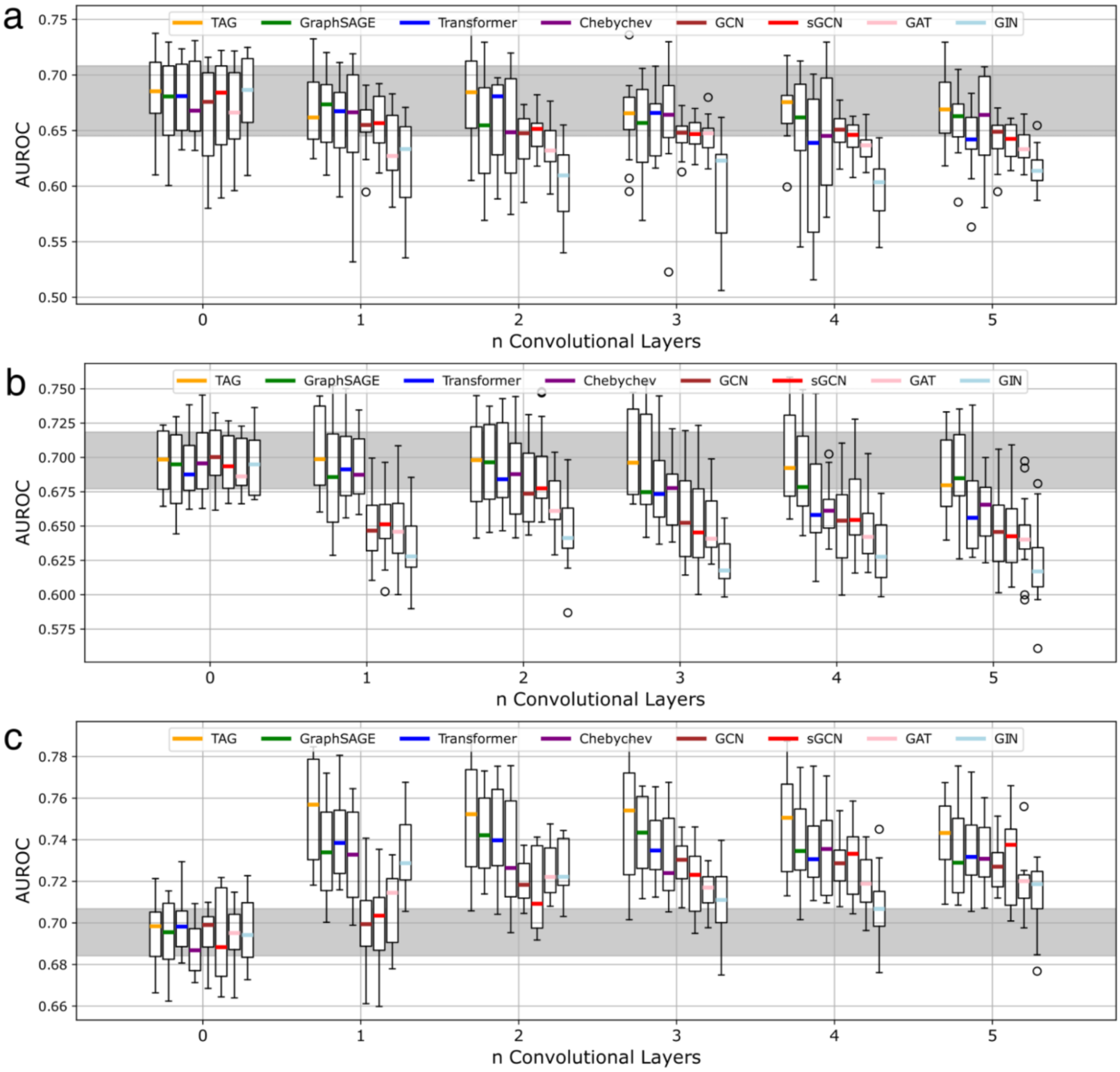

Supplementary Figure 6 | GNN Depth. Influence of the number of graph convolution layers r on model performance using the adjacency matrices a, BioPlex 3.0 HEK293T, b, GRNdb Adipose Tissue, c, IntAct Direct Interaction. Zero convolutional layers correspond to an MLP. The gray bar in the background denotes the interquartile range of all MLP-runs. Each boxplot is based on n = 16 values. Boxes represent the interquartile range, colored bars are medians, whiskers extend at most 1.5 times the interquartile range, and outliers are shown individual. The color coding indicates the type of GNN (see Supplementary Note 2 for more details).

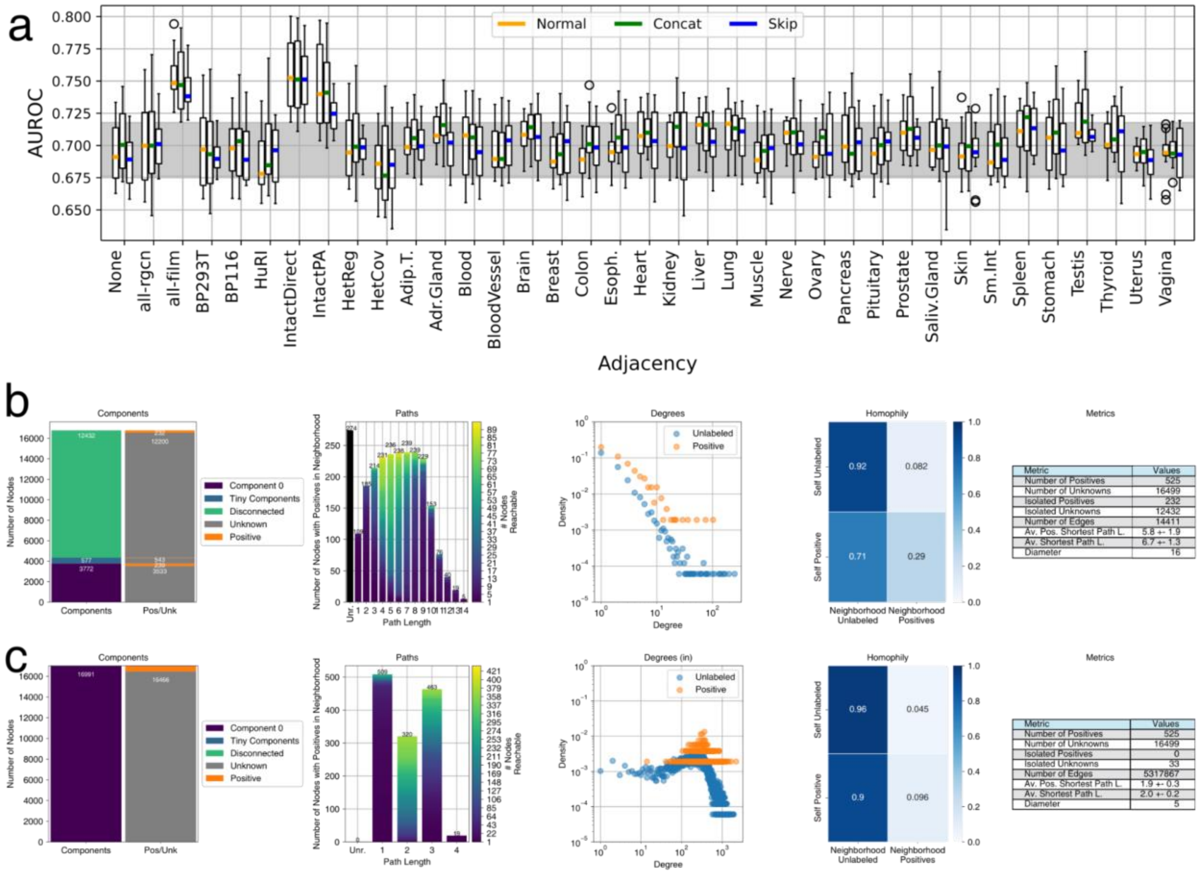

Supplementary Figure 7 | Network Performance and Properties. a, Boxplots of model performance (y-axis) for different adjacency matrices (x-axis). Adjacency “None” refers to an MLP that does not use any graph information. Boxes represent the interquartile range, colored bars are medians, whiskers extend at most 1.5 times the interquartile range, and outliers are shown individual. The gray bar in the background denotes the interquartile range of all MLP-runs. “Normal” indicates the normal information flow from pre-MP to MP to post-MP (Supplementary Figure 5c). “Concat” indicates that the output of pre-MP is concatenated to the output of MP before being passed into post-MP. “Skip” indicates that the output of pre-MP is added to the output of MP using a sum operation before being passed into post-MP. b, Network properties of IntAct Direct with the label set for immune dysregulation. c, Network properties of all networks merged together with the label set for immune dysregulation. Components: The left bar shows the fraction of the network that is either in the largest connected component (component 0), in microcomponents (smaller than 1% of all nodes), or isolated nodes which have no incident edge, right bar shows the distribution of labeled and unlabeled nodes. Paths: Each bar shows the number of positives which have other positives in the neighborhood of the indicated size. Color indicates the number of positives in the neighborhood for each node according to scale on the right. The black bar on the left indicates the number of isolated positives. Degrees: Degree distributions of positives and unlabeled nodes. Homophily: Plot shows the percentage of nodes in the neighborhood of a node that either share the same label or have the opposite label. Metrics: additional metrics of the graphs.

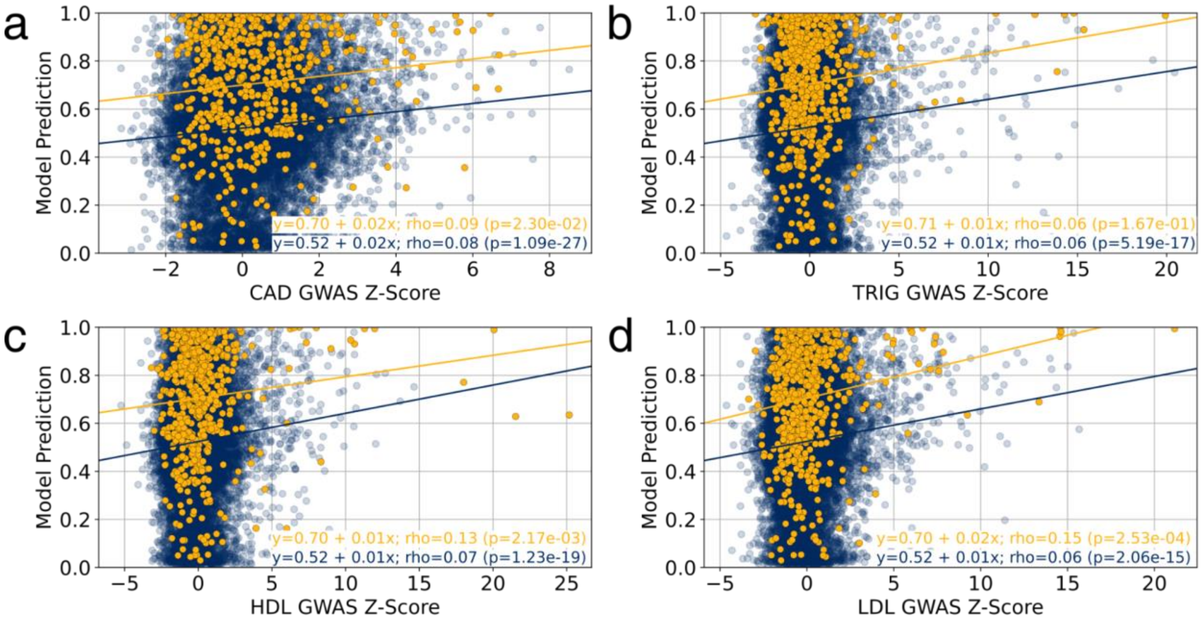

Supplementary Figure 8 | Relation of gene-level GWAS association and core gene prediction. Scatterplots show the relation between the model prediction of a single FiLM prediction model trained for cardiovascular disease (y-axis) and the gene-level association z-scores computed with MAGMA[87] (x-axis) from different GWAS studies: yellow points represent Mendelian disorder genes; blue points represent unlabeled genes. Predictions are obtained on the holdout set. a, coronary artery disease (CAD), b, triglyceride levels (TRIG), c, high density lipoprotein levels (HDL) and d, low density lipoprotein levels (LDL).

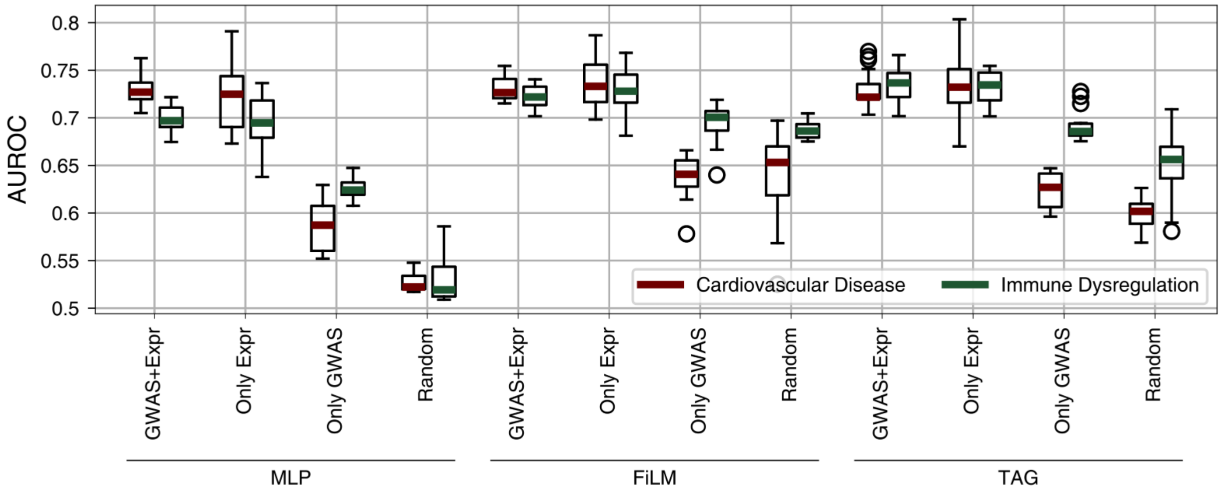

Supplementary Figure 9 | Input Feature Ablation Experiments. Three classifiers (MLP, no network information; FiLM, all networks; TAG, IntAct Direct Interaction) for cardiovascular disease and immune dysregulation are shown in an ablation experiment. Classifiers are either trained with full input features (GWAS+Expr.), without GWAS input features (Only Expr.), without gene expression features (Only GWAS) or with random input features (Random), leaving only the information of the biological network as background performance. Performance is evaluated on holdout sets (25%). While removing GWAS features mainly increases variance, removing gene expression features heavily impairs performance, but not as much as using random input features. We therefore conclude that, while GWAS is helpful and important, prediction performance for the majority of genes is rooted in gene expression patterns. Each boxplot is based on *n* = 16 values. Boxes represent the interquartile range, colored bars are medians, whiskers extend at most 1.5 times the interquartile range, and outliers are shown individually.

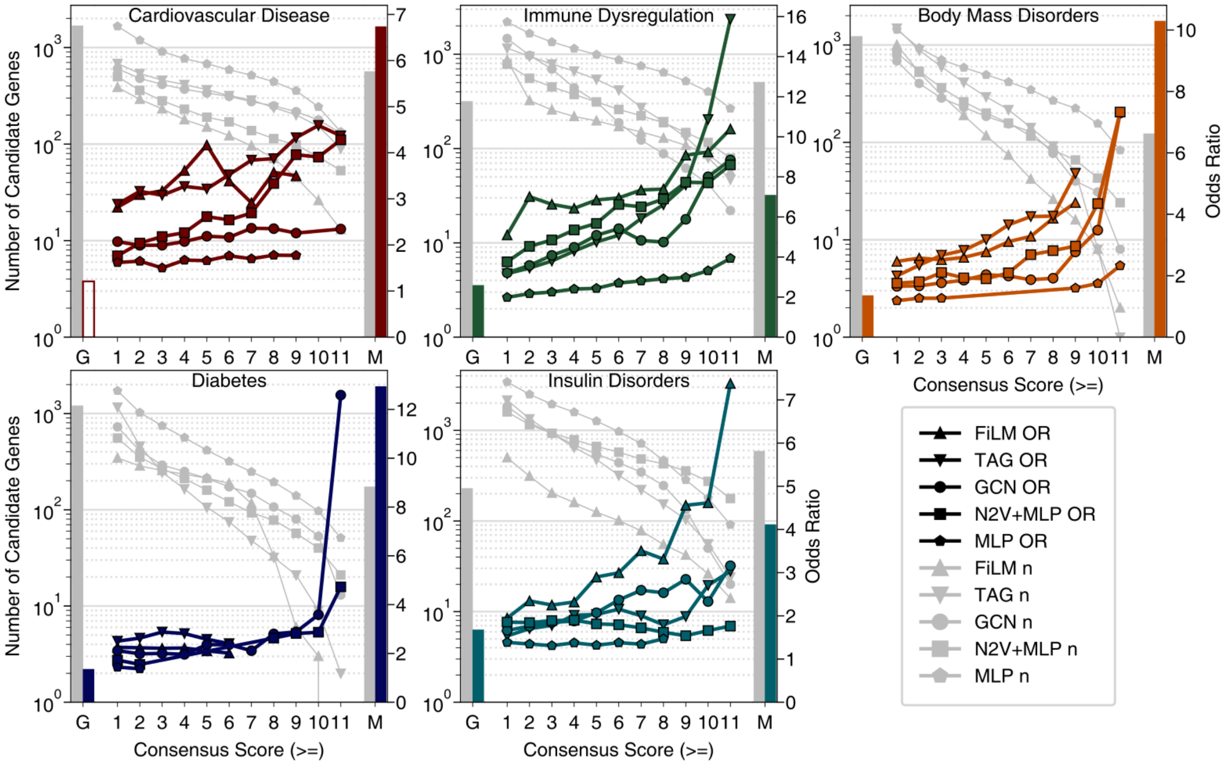

Supplementary Figure 10 | Mouse Knockout validation with GWAS Genes removed from Candidates. Odds ratio (OR) (right y-axis) for observing disease relevant phenotypes in mice with knockouts of orthologs of candidate core genes in the indicated convergence score bins (x-axis) of the five classifier methods (colored lines). Gray lines indicate strength of candidate gene sets (left y-axis) in the corresponding bin for the phenotypes as indicated in the panel. Only ORs with an FDR < 0.05 (Fisher's exact test) are shown. Bars to the right (M) and left (G) of each plot indicate set strength (gray) and OR (colored) of Mendelian genes and GWAS genes for each phenotype. Filled bars represent ORs with an FDR < 0.05, otherwise bars are hollow. Precise P-values, FDR, and n for each test are shown in Supplementary Data 3.

a

Method	Degree	CS ≥ 1 Candidates	CS ≥ 1 Non-Candidates	CS ≥ 1 p, OR	CS ≥ 8 Candidates	CS ≥ 8 Non-Candidates	CS ≥ 8 p, OR
FiLM	$d > 0$	186	4366	$p = 6.19\text{e}{-}08$	41	4511	$p = 3.24\text{e}{-}04$
	$d = 0$	299	11885	OR = 1.69	50	12134	OR = 2.21
TAG	$d > 0$	715	3837	$p = 8.99\text{e}{-}251$	276	4276	$p = 6.82\text{e}{-}122$
	$d = 0$	182	12002	OR = 12.3	30	12154	OR = 26.1
N2V+MLP	$d > 0$	218	4334	$p = 7.52\text{e}{-}03$	43	4509	$p = 5.48\text{e}{-}01$
	$d = 0$	469	11715	OR = 1.26	129	12055	OR = 0.89
FiLM	$d > 0$	168	4384	$p = 4.98\text{e}{-}02$	21	4531	$p = 9.00\text{e}{-}01$
Unbiased	$d = 0$	375	11809	OR = 1.21	59	12125	OR = 0.95

b

Method	Degree	CS ≥ 1 Candidates	CS ≥ 1 Non-Candidates	CS ≥ 1 p, OR	CS ≥ 8 Candidates	CS ≥ 8 Non-Candidates	CS ≥ 8 p, OR
FiLM	$d > 0$	665	3771	$p = 1.58\text{e}{-}160$	143	4293	$p = 2.47\text{e}{-}60$
	$d = 0$	340	11723	OR = 6.08	20	12043	OR = 20.1
TAG	$d > 0$	1277	3159	$p = 0.00$	212	4224	$p = 2.67\text{e}{-}123$
	$d = 0$	0	12063	OR = inf	0	12063	OR = inf
N2V+MLP	$d > 0$	348	4064	$p = 2.52\text{e}{-}16$	103	4309	$p = 3.22\text{e}{-}14$
	$d = 0$	536	11381	OR = 1.82	93	11824	OR = 3.04
FiLM	$d > 0$	238	4198	$p = 5.76\text{e}{-}14$	72	4364	$p = 3.87\text{e}{-}10$
Unbiased	$d = 0$	341	11722	OR = 1.95	66	11997	OR = 3.0

Supplementary Figure 11 | Biased results from aggregating small-scale Literature. a, shown are the 2 x 2 contingency tables of candidates and noncandidates with two different Consensus score (CS) cutoffs for cardiovascular disease and their involvement in the IntAct Direct Interaction network. Degree $d > 0$ denotes genes that have at least one incident edge in IntAct Direct Interaction, while $d = 0$ denotes genes that are isolated in IntAct Direct Interaction. Odds Ratios (OR) and *P*-values obtained via Fisher's exact test, unadjusted. b, shown are the 2 x 2 contingency tables of candidates and noncandidates with two different Consensus score (CS) cutoffs for immune dysregulation and their involvement in the IntAct Direct Interaction network. Degree $d > 0$ denotes genes that have at least one incident edge in IntAct Direct Interaction, while $d = 0$ denotes genes that are isolated in IntAct Direct Interaction. Odds Ratios (OR) and *P*-values obtained via Fisher's exact test, unadjusted.

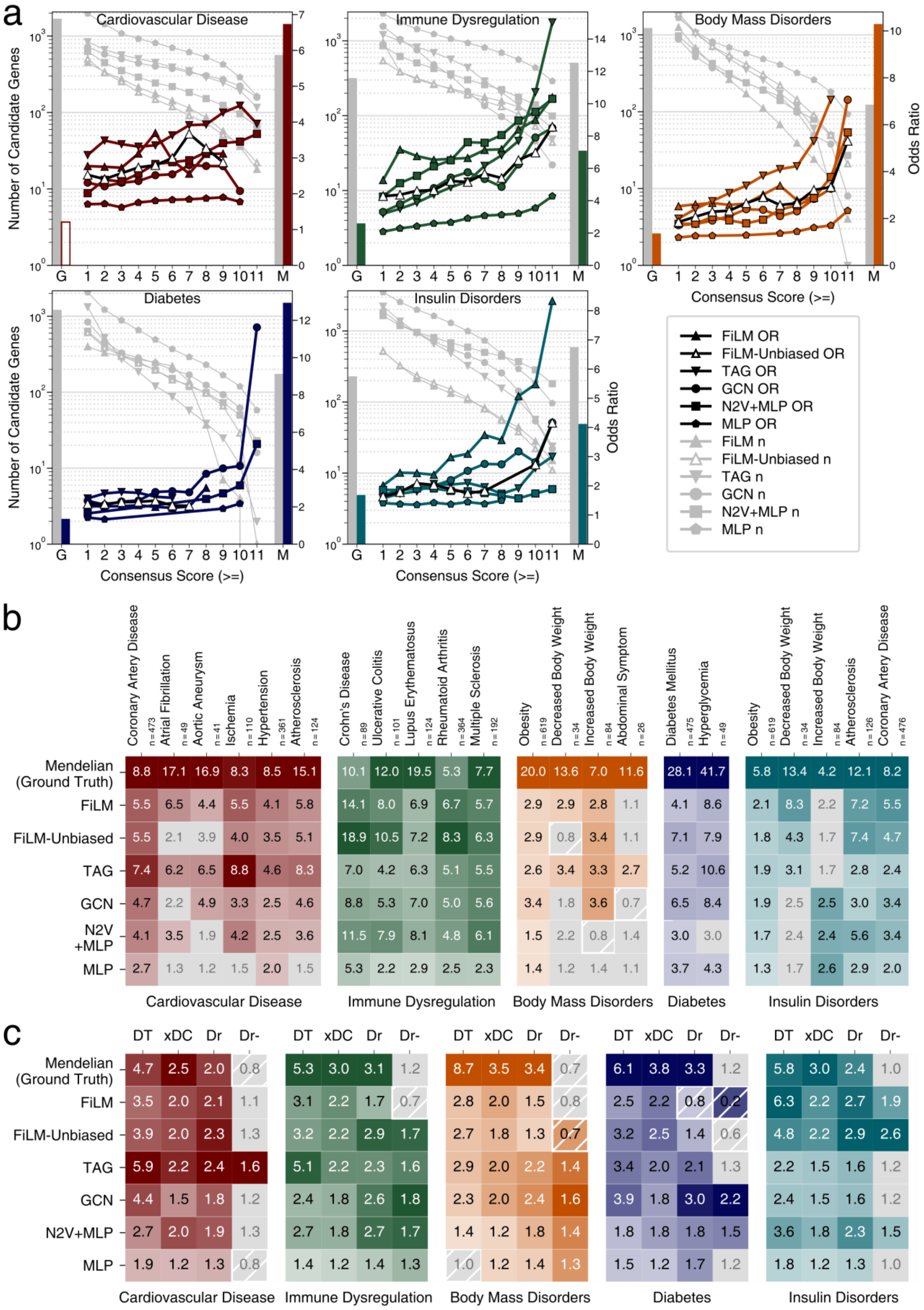

Supplementary Figure 12 | Unbiased external validation. a, Odds ratio (OR) (right y-axis) for observing disease relevant phenotypes in mice with knock-outs of orthologs of candidate core genes

in the indicated consensus score bins (x-axis) of the five classifier methods (colored lines). Gray lines indicate strength of candidate gene sets (left y-axis) in the corresponding bin for the phenotypes as indicated in the panel. Only ORs with an FDR < 0.05 are shown. Bars to the right of each plot (M) indicate set strength (gray) and OR (colored) of Mendelian genes for each phenotype. Precise *P*-values, FDR, and n for each test are shown in Supplementary Data 4. b, Odds ratios (ORs) of Mendelian genes (first row) and of candidate genes of the five selected methods (rows) for common complex subtypes of the five Mendelian disorder groups. ORs with a FDR > 0.05 in gray. c, Enrichment of drug targets and druggability in Mendelian disorder genes and indicated candidate gene sets. DT: OR of known drug targets. xDC: Ratio of median number of drug-gene interactions per candidate gene to the median of non-candidates, only genes with drug-gene interactions are considered. Dr: OR of druggable genes. Dr-: OR of druggable genes, after all drug targets have been removed. Odds Ratios with FDR > 0.05 are grayed out. For all panels, precise *P*-values, FDR, and n for each test are shown in Supplementary Data 9 & 23.

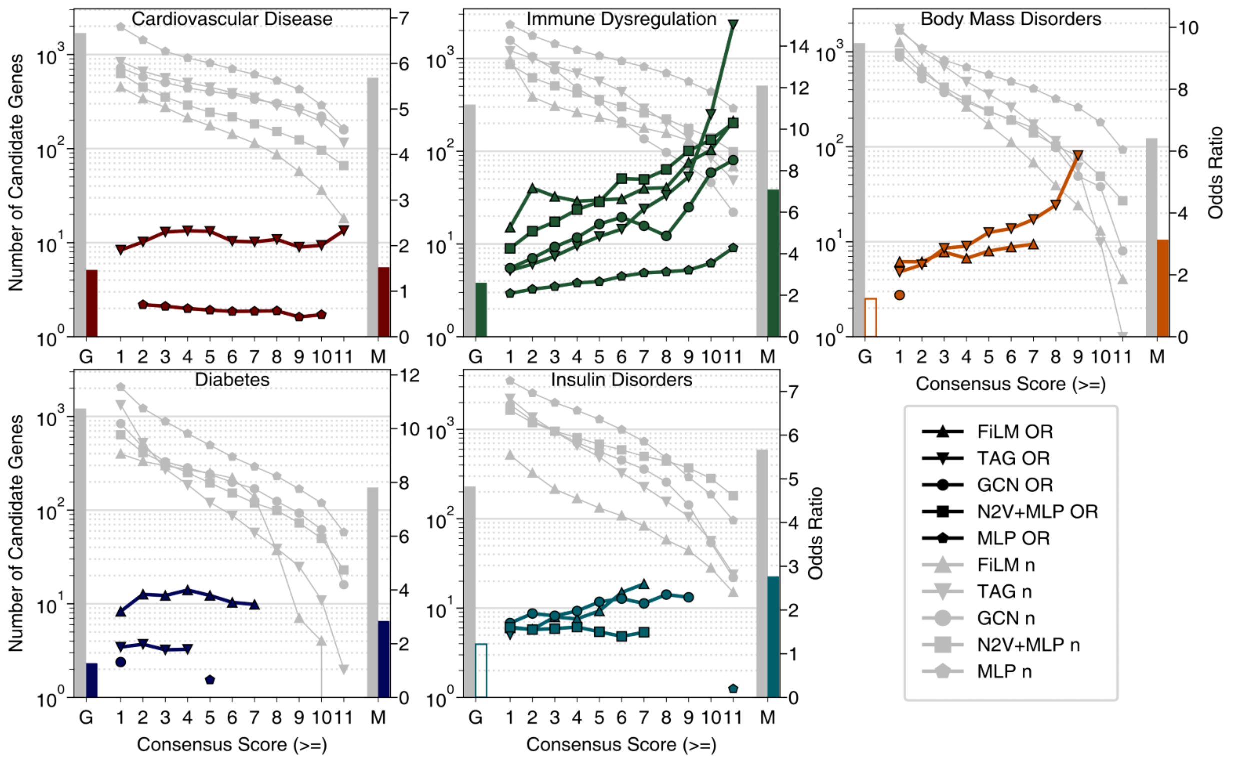

Supplementary Figure 13 | Mouse Knockout validation disease-specificity experiments. Candidate genes for all five disorders are validated against the mouse knockout genes for immune dysregulation. Odds ratio (OR) (right y-axis) for observing disease relevant phenotypes in mice with knockouts of orthologs of candidate core genes in the indicated convergence score bins (x-axis) of the five classifier methods (colored lines). Gray lines indicate strength of candidate gene sets (left y-axis) in the corresponding bin for the phenotypes as indicated in the panel. Only ORs with an FDR < 0.05 (Fisher's exact test) are shown. Bars to the right (M) and left (G) of each plot indicate set strength (gray) and OR (colored) of Mendelian genes and GWAS genes for each phenotype. Filled bars represent ORs with an FDR < 0.05, otherwise bars are hollow. Precise P-values, FDR, and n for each test are shown in Supplementary Data 4.

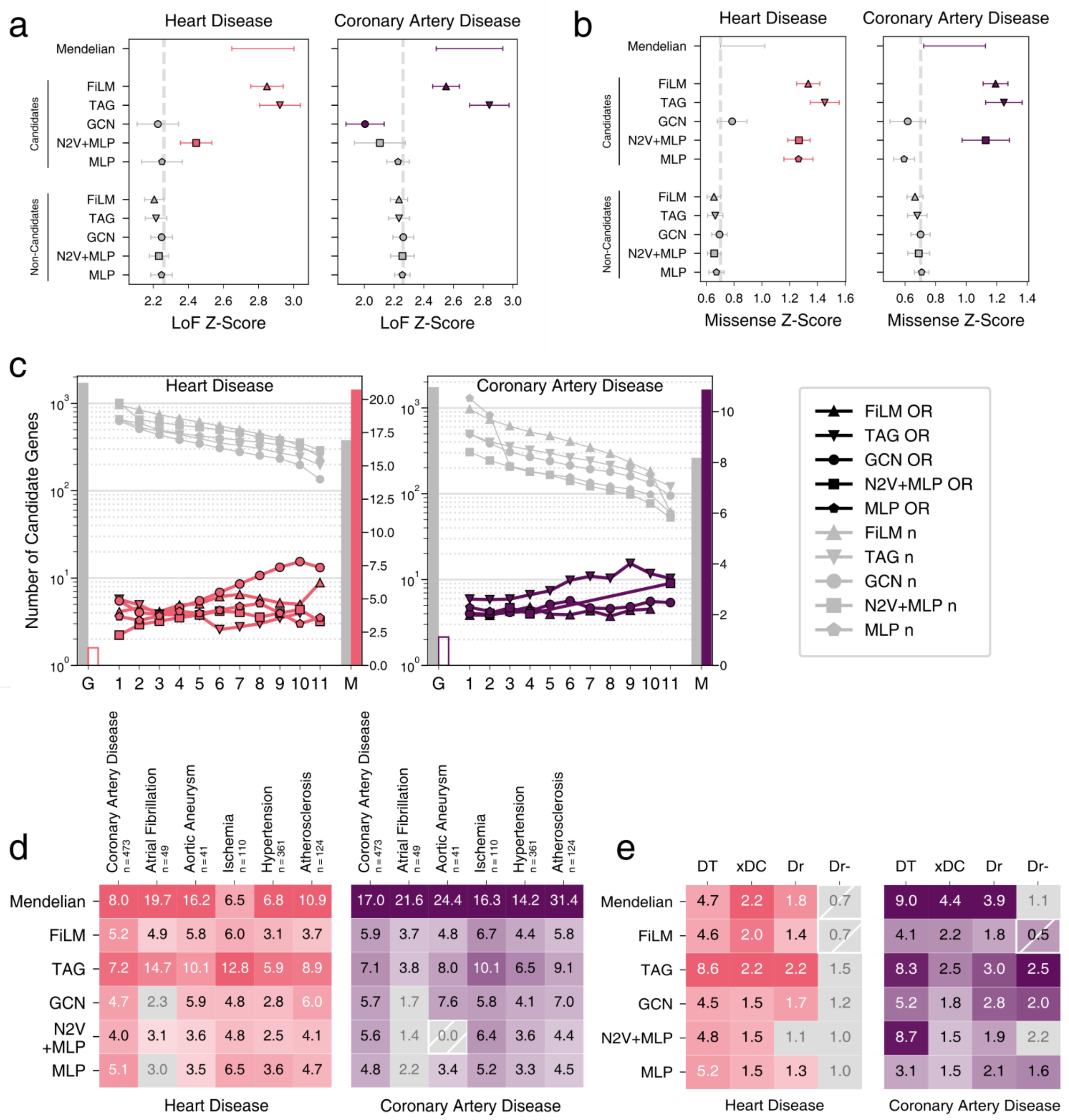

Supplementary Figure 14 | External Validation of Novel Phenotypes. a, b, LoF intolerance and missense mutation intolerance Z-scores of Mendelian genes, and the indicated candidate and non-candidate sets generated by the five methods. Shown are group means and 95% confidence intervals of Tukey's HSD test. Colored symbols and error bars indicate P < 0.05 in comparison with respective non-candidate sets; not significant sets in gray. Dashed line indicates the mean across all genes. c, Odds ratio (OR) (right y-axis) for observing disease relevant phenotypes in mice with knockouts of orthologs of candidate core genes in the indicated convergence score bins (x-axis) of the five classifier methods (colored lines). Gray lines indicate strength of candidate gene sets (left y-axis) in the corresponding bin for the phenotypes as indicated in the panel. Only ORs with an FDR < 0.05 (Fisher's exact test) are shown. Bars to the right (M) and left (G) of each plot indicate set strength (gray) and OR (colored) of Mendelian genes and GWAS genes for each phenotype. Bars representing significant ORs are filled, hollow bars represent non-significant ORs. d, Odds ratios (ORs) of Mendelian genes (first row) and of candidate genes of the five selected methods (rows) for common complex subtypes of the Mendelian disorder subgroups. ORs with FDR > 0.05 (Fisher's exact test) in gray. e, Enrichment of drug targets and druggability in Mendelian disorder genes and

indicated candidate gene sets. DT: OR of known drug targets. xDC: Ratio of median number of drug-gene interactions per candidate gene to the median of non-candidates, only genes with drug-gene interactions are considered. Ratios with FDR > 0.05 (U-test) are grayed out. Dr: OR of druggable genes. Dr-: OR of druggable genes, after all drug targets have been removed. Odds Ratios with FDR > 0.05 (Fisher's exact test) are grayed out. Precise P-values, FDR, and n for each test in each panel are shown in Supplementary Data 16 - 29, respectively.

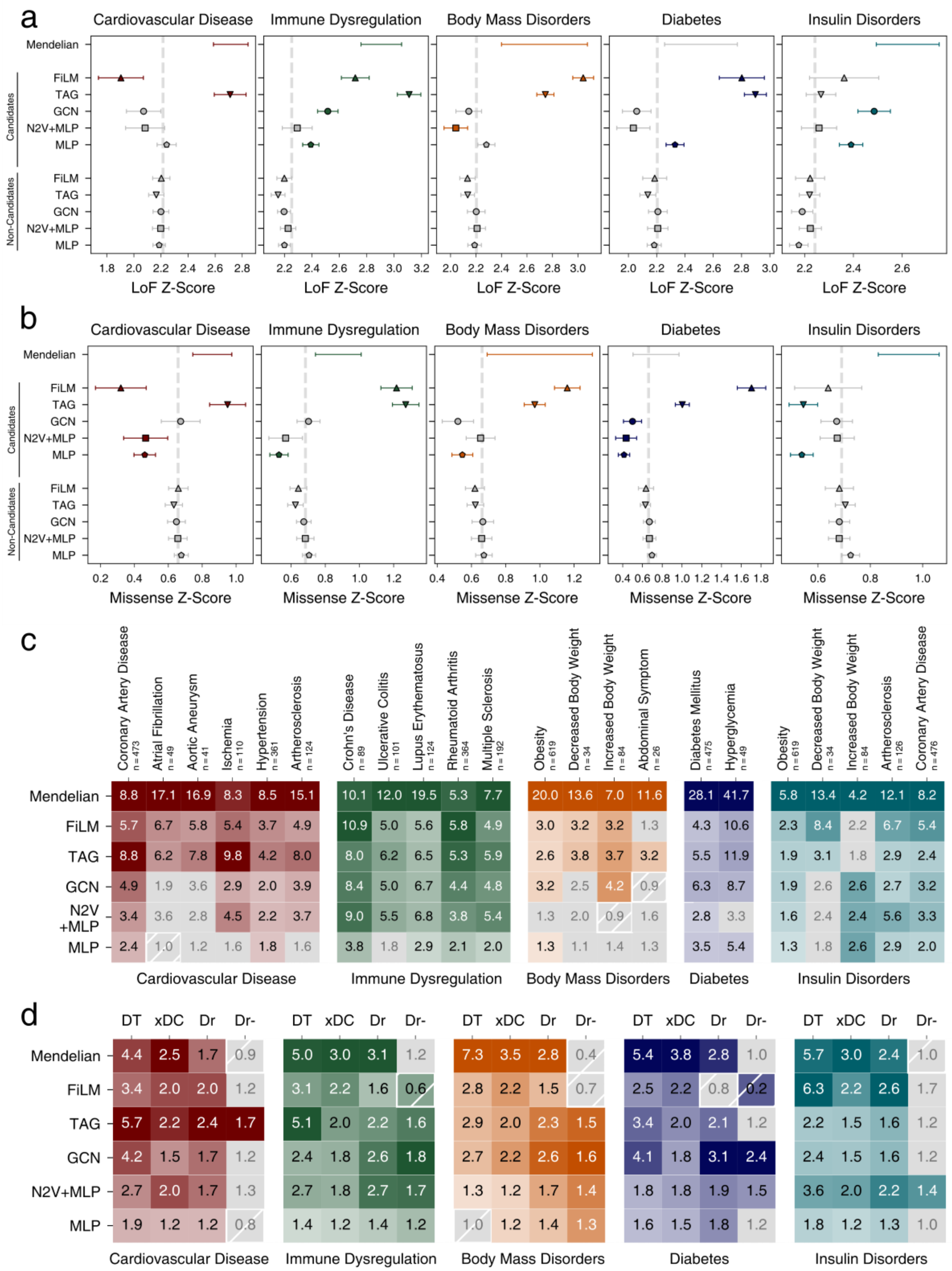

Supplementary Figure SF15 | External Validation with GWAS genes removed from Candidates. a, b, LoF intolerance and missense mutation intolerance Z-scores of Mendelian genes, and the indicated candidate and non-candidate sets generated by the five methods. Shown are group means and 95% confidence intervals of Tukey's HSD test. Colored symbols and error bars indicate FDR < 0.05 in comparison with respective non-candidate sets; not significant sets in gray. Dashed line indicates the mean across all genes. c, Odds ratios (ORs) of Mendelian genes (first row) and of

candidate genes of the five selected methods (rows) for common complex subtypes of the Mendelian disorder subgroups. ORs with FDR > 0.05 (Fisher's exact test) in gray. d, Enrichment of drug targets and druggability in Mendelian disorder genes and indicated candidate gene sets. DT: OR of known drug targets. xDC: Ratio of median number of drug-gene interactions per candidate gene to the median of non-candidates, only genes with drug-gene interactions are considered. Ratios with FDR > 0.05 (U-test) are grayed out. Dr: OR of druggable genes. Dr-: OR of druggable genes, after all drug targets have been removed. Odds Ratios with FDR > 0.05 (Fisher's exact test) are grayed out. Precise P-values, FDR, and n for each test in each panel are shown in Supplementary Data 10 - 13, respectively.

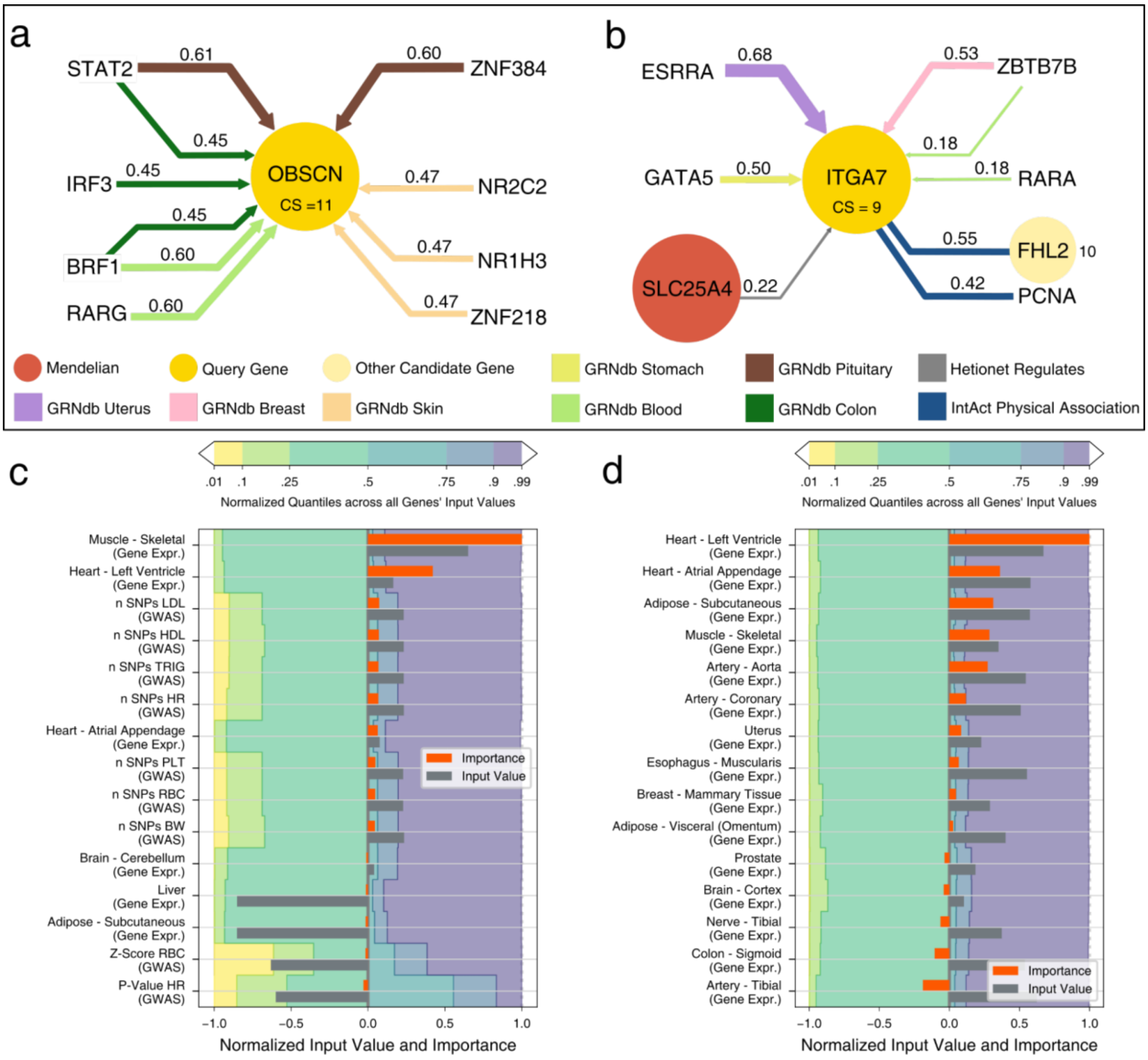

Supplementary Figure 16 | Model Interpretation. a. Most important edges for FiLM's prediction of OBSCN as candidate gene for cardiovascular disease. Shown are HGNC gene symbols. The query gene node is shown in the center, with adjacent nodes connected by relevant edges in the periphery. Candidate genes are signed with their Consensus Score (CS). The color of the edges denotes the network and the strength of the edge shows the relative importance for the prediction of the query gene which is also written at the edge. Arrowheads indicate direction of edges, undirected edges have no arrowheads. A value of 1 means that it is the most important edge for all models of the ensemble, while a value of 0 indicates that it is the least important edge for every model. Shown are 10 out of 4.5 million edges, 312 of which are in the direct neighborhood of the query gene. b. Most important edges for FiLM's prediction of ITGA7 as candidate gene for cardiovascular disease. Shown are 8 out of 4.5 million edges, 316 of which are in the direct neighborhood of the query gene. c,d: Input feature importance for OBSCN and ITGA7 alongside the respective feature's input value, compared to the input values of other genes by the quantile borders in the background. Shown are the 10 features with the strongest positive influence and the 5 features with the strongest negative influence. Negative input values are normalized to the interval [-1; 0] and positive input values to (0 ; 1] for visualization. Gray bars exceeding the colored areas are either below the 1% quantile or above the 99% quantile of that input feature. Importance values are obtained by integrated gradients and normalized to the interval [-1; 1]. Positive importance values are in favor of the prediction as candidate genes, negative importance values are attributed to features that contradict the prediction.

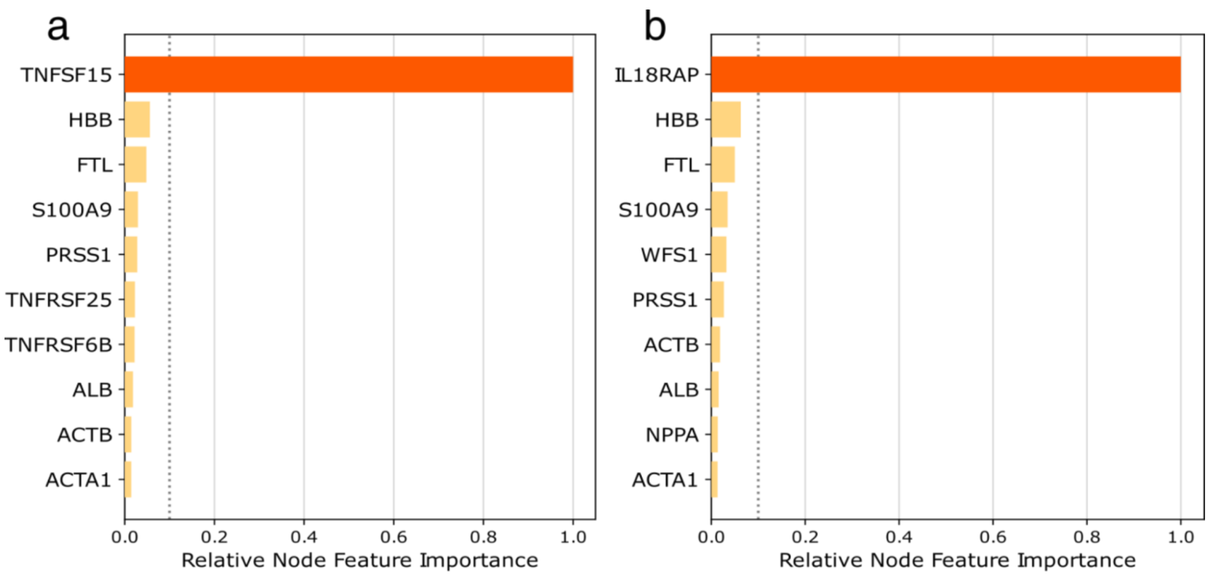

Supplementary Figure 17 | Neighborhood Feature Importance. Shown is the cumulative normalized importance of a node's input features for the prediction of query nodes a: TNFSF15 and b: IL18RAP as candidate genes for immune dysregulation predicted by FiLM. Shown are the top 10 most influential nodes. A node that has the most relevant input features for the prediction of the query nodes every for model in the ensemble has a value of 1, while a node that has the least relevant input features for every model has a value of 0. The dashed gray line denotes the threshold of 0.1.

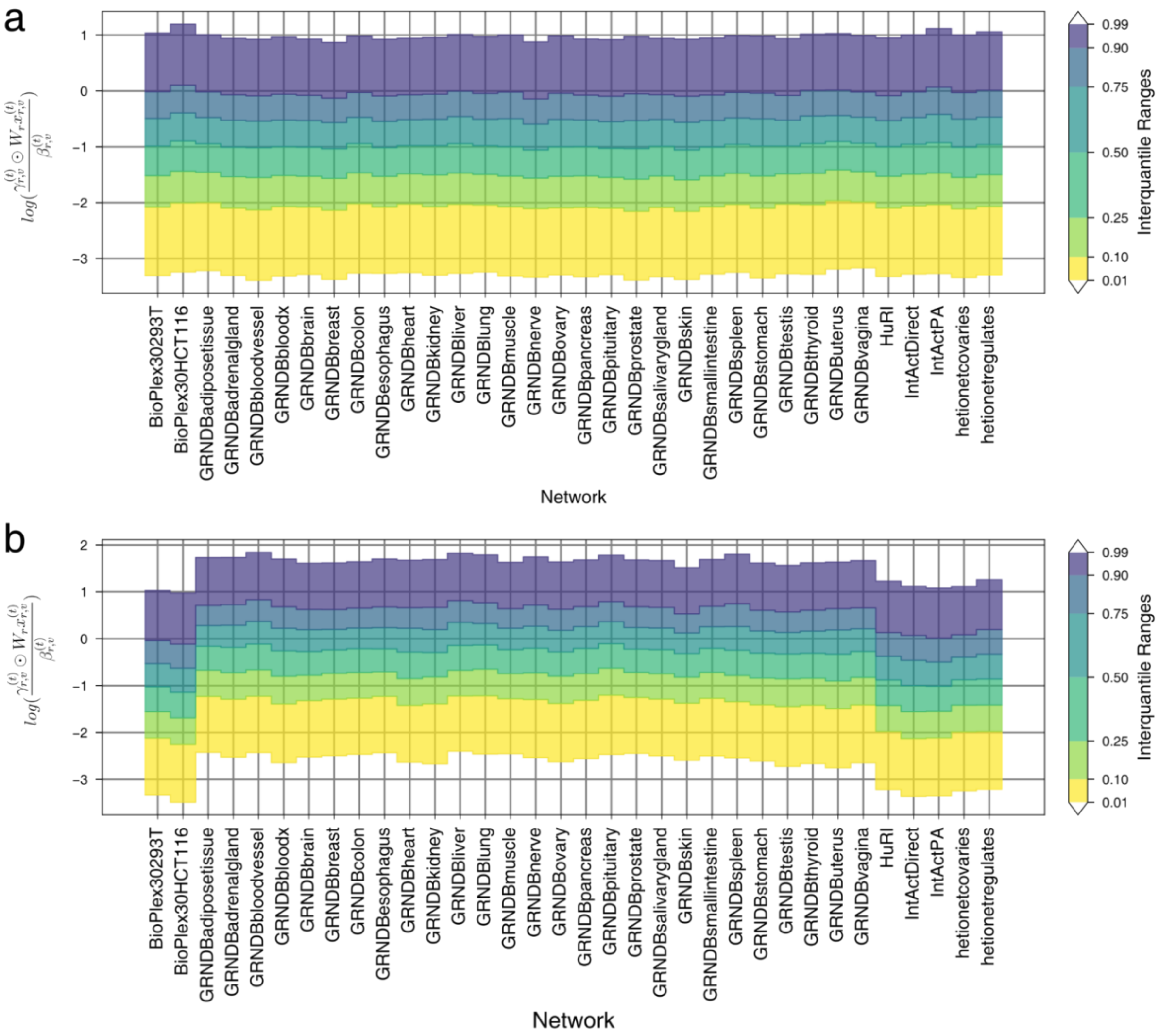

Supplementary Figure 18 | Ratio of Sender to Receiver features. Shown is the influence of the bias term on the features of the messages passed along the edges of all networks in a single FiLM model trained for immune dysregulation. a, values for the first layer. b shows the values for the second layer. Low values indicate that the message is dominated by the bias term and thus independent of the sender's features.

Supplementary References

1. Luck, K. *et al.* A reference map of the human binary protein interactome. *Nature* **580**, 402–408 (2020).

2. Huttlin, E. L. *et al.* Dual Proteome-scale Networks Reveal Cell-specific Remodeling of the Human Interactome. *Cell* **184**, 3022-3040.e28 (2021).

3. Yu, H. *et al.* High Quality Binary Protein Interaction Map of the Yeast Interactome Network. *Science* **322**, 104–110 (2008).

4. Edwards, A. M. *et al.* Too many roads not taken. *Nature* **470**, 163–165 (2011).

5. Zhu, J. *et al.* Beyond Homophily in Graph Neural Networks: Current Limitations and Effective Designs. *ArXiv200611468 Cs Stat* (2020).

6. Orchard, S. *et al.* The MIntAct project—IntAct as a common curation platform for 11 molecular interaction databases. *Nucleic Acids Res.* **42**, D358–D363 (2014).

7. Szklarczyk, D. *et al.* The STRING database in 2021: customizable protein-protein networks, and functional characterization of user-uploaded gene/measurement sets. *Nucleic Acids Res.* **49**, D605–D612 (2021).

8. Du, J., Zhang, S., Wu, G., Moura, J. M. F. & Kar, S. Topology Adaptive Graph Convolutional Networks. Preprint at http://arxiv.org/abs/1710.10370 (2018).

9. Hamilton, W. L., Ying, R. & Leskovec, J. Inductive Representation Learning on Large Graphs. *ArXiv170602216 Cs Stat* (2018).

10. Shi, Y. *et al.* Masked Label Prediction: Unified Message Passing Model for Semi-Supervised Classification. Preprint at https://doi.org/10.48550/arXiv.2009.03509 (2021).

11. Defferrard, M., Bresson, X. & Vandergheynst, P. Convolutional Neural Networks on Graphs with Fast Localized Spectral Filtering. Preprint at https://doi.org/10.48550/arXiv.1606.09375 (2017).

12. Kipf, T. N. & Welling, M. Semi-Supervised Classification with Graph Convolutional Networks. *ArXiv160902907 Cs Stat* (2016).

13. Wu, F. *et al.* Simplifying Graph Convolutional Networks. Preprint at https://doi.org/10.48550/arXiv.1902.07153 (2019).

14. Veličković, P. *et al.* Graph Attention Networks. *ArXiv171010903 Cs Stat* (2017).

15. Xu, K., Hu, W., Leskovec, J. & Jegelka, S. How Powerful are Graph Neural Networks? *ArXiv181000826 Cs Stat* (2019).

16. Schlichtkrull, M. *et al.* Modeling Relational Data with Graph Convolutional Networks. *ArXiv170306103 Cs Stat* (2017).

17. Busbridge, D., Sherburn, D., Cavallo, P. & Hammerla, N. Y. Relational Graph Attention Networks. *ArXiv190405811 Cs Stat* (2019).

18. Brockschmidt, M. GNN-FiLM: Graph Neural Networks with Feature-wise Linear Modulation. Preprint at http://arxiv.org/abs/1906.12192 (2020).

19. Zhang, S.-W., Xu, J.-Y. & Zhang, T. DGMP: Identifying Cancer Driver Genes by Jointing DGCN and MLP from Multi-Omics Genomic Data. 2022.02.16.480791 Preprint at https://doi.org/10.1101/2022.02.16.480791 (2022).

20. Gilmer, J., Schoenholz, S. S., Riley, P. F., Vinyals, O. & Dahl, G. E. Neural message passing for Quantum chemistry. in *Proceedings of the 34th International Conference on Machine Learning - Volume 70* 1263–1272 (JMLR.org, 2017).

21. Yang, Y. & Li, D. NENN: Incorporate Node and Edge Features in Graph Neural Networks. in *Proceedings of The 12th Asian Conference on Machine Learning* 593–608 (PMLR, 2020).

22. Chamberlain, B. P. *et al.* GRAND: Graph Neural Diffusion. Preprint at https://doi.org/10.48550/arXiv.2106.10934 (2021).

23. Horn, M. *et al.* Topological Graph Neural Networks. Preprint at https://doi.org/10.48550/arXiv.2102.07835 (2022).

24. Freund, M. K. *et al.* Phenotype-Specific Enrichment of Mendelian Disorder Genes near GWAS Regions across 62 Complex Traits. *Am. J. Hum. Genet.* **103**, 535–552 (2018).

25. Cunningham, F. *et al.* Ensembl 2022. *Nucleic Acids Res.* **50**, D988–D995 (2022).

26. Randazzo, D., Pierantozzi, E., Rossi, D. & Sorrentino, V. The potential of obscurin as a therapeutic target in muscle disorders. *Expert Opin. Ther. Targets* **21**, 897–910 (2017).

27. Arimura, T. *et al.* Structural analysis of obscurin gene in hypertrophic cardiomyopathy. *Biochem. Biophys. Res. Commun.* **362**, 281–287 (2007).

28. Grogan, A. *et al.* Deletion of obscurin immunoglobulin domains Ig58/59 leads to age-dependent cardiac remodeling and arrhythmia. *Basic Res. Cardiol.* **115**, 60 (2020).

29. Lagor, W. R. *et al.* Genetic manipulation of the ApoF/Stat2 locus supports an important role for Type I Interferon signaling in atherosclerosis. *Atherosclerosis* **233**, 234–241 (2014).

30. Lee, C.-J. *et al.* Stat2 stability regulation: an intersection between immunity and carcinogenesis. *Exp. Mol. Med.* **52**, 1526–1536 (2020).

31. Liu, S. *et al.* ZNF384: A Potential Therapeutic Target for Psoriasis and Alzheimer's Disease

Through Inflammation and Metabolism. *Front. Immunol.* **13**, 892368 (2022).

32. Desroches-Castan, A., Cherradi, N., Feige, J.-J. & Ciais, D. A novel function of Tis11b/BRF1 as a regulator of Dll4 mRNA 3′-end processing. *Mol. Biol. Cell* **22**, 3625–3633 (2011).

33. King, K. R. *et al.* IRF3 and type I interferons fuel a fatal response to myocardial infarction. *Nat. Med.* **23**, 1481–1487 (2017).

34. Bhandari, A. *et al.* ITGA7 functions as a tumor suppressor and regulates migration and invasion in breast cancer. *Cancer Manag. Res.* **10**, 969–976 (2018).

35. Zhang, X., Ke, S., Lu, Y. & An, H. ITGA7 relates to disease risk, pathological feature, treatment response and survival in Ph- acute lymphoblastic leukemia. *Biomark. Med.* **15**, 1589–1597 (2021).

36. Xia, W. *et al.* Case Report: A Boy From a Consanguineous Family Diagnosed With Congenital Muscular Dystrophy Caused by Integrin Alpha 7 (ITGA7) Mutation. *Front. Genet.* **12**, 706823 (2021).

37. Bugiardini, E. *et al.* Integrin α7 Mutations Are Associated With Adult-Onset Cardiac Dysfunction in Humans and Mice. *J. Am. Heart Assoc.* **11**, e026494 (2022).

38. Burkin, D. J. *et al.* Transgenic expression of {alpha}7{beta}1 integrin maintains muscle integrity, increases regenerative capacity, promotes hypertrophy, and reduces cardiomyopathy in dystrophic mice. *Am. J. Pathol.* **166**, 253–263 (2005).

39. Esposito, T. *et al.* Digenic mutational inheritance of the integrin alpha 7 and the myosin heavy chain 7B genes causes congenital myopathy with left ventricular non-compact cardiomyopathy. *Orphanet J. Rare Dis.* **8**, 91 (2013).

40. Argirò, A. *et al.* Sex-Related Differences in Genetic Cardiomyopathies. *J. Am. Heart Assoc.* **11**, e024947 (2022).

41. Rowland, T. J. *et al.* Obscurin Variants in Patients With Left Ventricular Noncompaction. *J. Am. Coll. Cardiol.* **68**, 2237–2238 (2016).

42. Marston, S. *et al.* OBSCN Mutations Associated with Dilated Cardiomyopathy and Haploinsufficiency. *PLOS ONE* **10**, e0138568 (2015).

43. Ramlawi, B., Abu Saleh, W. K. & Edgerton, J. The Left Atrial Appendage: Target for Stroke Reduction in Atrial Fibrillation. *Methodist DeBakey Cardiovasc. J.* **11**, 100–103 (2015).
