# Supplementary material for: Speos: an ensemble graph representation learning framework to predict core gene candidates for complex diseases
Source: Nat Commun. 2023 Nov 8;14:7206. doi: 10.1038/s41467-023-42975-z (PMC10632370; doi:10.1038/s41467-023-42975-z)
Supplement: Supplementary file 3 — Description of Additional Supplementary Files [file 41467_2023_42975_MOESM3_ESM.pdf]

Description of Additional Supplementary Files

File Name: Supplementary Data 1
Description: Ensemble Concordance. Each outer fold's predictions are evaluated for concordance among the models. Finally, bins with significantly larger concordance in held out positives than expected by chance are selected.

File Name: Supplementary Data 2
Description: Mouse KO Experiments. Contingency tables of candidate and Mendelian genes, respectively, against the background set, odds ratio, and the results of the Fisher's exact test (two-sided).

File Name: Supplementary Data 3
Description: Mouse KO Experiments without GWAS. Contingency tables of candidates/Mendelian genes against the background set, odds ratio and the results of the Fisher's exact test (two-sided). GWAS genes identified from the input GWAS ($p<5e-8$) are removed from the candidates.

File Name: Supplementary Data 4
Description: Mouse KO Experiments including FiLM-Unbiased. Contingency tables of candidates and Mendelian genes, respectively, against the background set, odds ratio, and the results of the Fisher's exact test (two-sided).

File Name: Supplementary Data 5
Description: Mouse Knockout validation disease-specificity experiments. Contingency tables of candidates/Mendelian genes against the background set, odds ratio and the results of the Fisher's exact test (two-sided). Each set of candidates/Mendelians is validated against the mouse knockout genes of immune dysregulation to demonstrate disease specificity.

File Name: Supplementary Data 6
Description: Biological Processes. Gene set enrichment analysis for gene ontology (GO) biological process using Fisher's exact tests (one-sided).

File Name: Supplementary Data 7
Description: Comparison of Mendelian and Candidate Log Odds. Log odds of the highest significant bin of candidate genes vs. the log odds of the Mendelian disorder genes using a Z-test (two-sided).

File Name: Supplementary Data 8
Description: Differentially Expressed Genes. Contingency tables of differentially expressed candidates/Mendelian genes against the background set, the enrichment odds ratio and the results of the Fisher's exact tests (two-sided).

File Name: Supplementary Data 9
Description: Differentially Expressed Genes including FiLM-Unbiased. Contingency tables of differentially expressed candidates/Mendelian genes against the background set, the enrichment odds ratio and the results of the Fisher's exact tests (two-sided).

File Name: **Supplementary Data 10**
Description: **Loss of Function (Lof) Mutation intolerance without GWAS Genes.** Group-wise sample sizes, means, 95% confidence intervals and the results of pairwise group comparisons using Tukey's HSD (two-sided).

File Name: **Supplementary Data 11**
Description: **Missense Mutation Intolerance without GWAS Genes.** Group-wise sample sizes, means, 95% confidence intervals and the results of pairwise group comparisons using Tukey's HSD (two-sided).

File Name: **Supplementary Data 12**
Description: **Differentially Expressed Genes without GWAS Genes.** Contingency tables of differentially expressed candidates/Mendelian genes against the background set, the enrichment odds ratio and the results of the Fisher's exact tests (two-sided).

File Name: **Supplementary Data 13**
Description: **Drug Target Analysis without GWAS Genes.** Enrichment of drug targets, number of drugs targeting each gene and enrichment of druggable genes among Mendelian disorder genes and candidate genes.

File Name: **Supplementary Data 14**
Description: **Loss of Function (Lof) Mutation intolerance.** Group-wise sample sizes, means, 95% confidence intervals and the results of pairwise group comparisons using Tukey's HSD (two-sided).

File Name: **Supplementary Data 15**
Description: **Missense Mutation intolerance.** Group-wise sample sizes, means, 95% confidence intervals and the results of pairwise group comparisons using Tukey's HSD (two-sided).

File Name: **Supplementary Data 16**
Description: **Loss of Function (Lof) Mutation intolerance for two new Phenotypes** Group-wise sample sizes, means, 95% confidence intervals and the results of pairwise group comparisons using Tukey's HSD (two-sided).

File Name: **Supplementary Data 17**
Description: **Missense Mutation Intolerance For two new Phenotypes.** Group-wise sample sizes, means, 95% confidence intervals and the results of pairwise group comparisons using Tukey's HSD (two-sided).

File Name: **Supplementary Data 18**
Description: **Mouse KO Experiments for two new Phenotypes.** Contingency tables of candidates and Mendelian genes, respectively, against the background set, odds ratio, and the results of the Fisher's exact test (two-sided).

File Name: **Supplementary Data 19**

Description: **Differentially Expressed Genes for two new Phenotypes.** Contingency tables of differentially expressed candidates/Mendelian genes against the background set, the enrichment odds ratio and the results of the Fisher's exact tests (two-sided).

File Name: **Supplementary Data 20**
Description: **Drug Target Analysis for two new Phenotypes.** Enrichment of drug targets, number of drugs targeting each gene and enrichment of druggable genes among Mendelian disorder genes and candidate genes.

File Name: **Supplementary Data 21**
Description: **Queries for two Novel Phenotypes.** These queries have been used to obtain new Mendelian disorder gene lists and the respective mouse knockout genes for validation. To reproduce the mouse knockout sets for the respective disease, go to http://www.informatics.jax.org/allele, select "Targeted" from "Generation Method", "Null/knockout" from "Allele Attributes" and click "Search".

File Name: **Supplementary Data 22**
Description: **Drug Target Analysis.** Enrichment of drug targets, number of drugs targeting each gene and enrichment of druggable genes among Mendelian disorder genes and candidate genes.

File Name: **Supplementary Data 23**
Description: **Drug Target Analysis including FiLM-Unbiased.** Enrichment of drug targets, number of drugs targeting each gene and enrichment of druggable genes among Mendelian disorder genes and candidate genes.

File Name: **Supplementary Data 24**
Description: **Results Summary.** Each gene is individually listed with detailed results, i.e. consensus score of the individual methods, results of external validations.

File Name: **Supplementary Data 25**
Description: **Disease to GWAS mappings.** Each disease is mapped to a selection of GWAS traits. The summary statistics of all selected GWAS traits are aggregated to gene level and used as input features in addition to tissue-specific gene expression features.

File Name: **Supplementary Data 26**
Description: **Mouse Knockout Queries.** To reproduce the mouse knockout sets for the respective disease, go to http://www.informatics.jax.org/allele, select "Targeted" from "Generation Method", "Null/knockout" from "Allele Attributes" and click "Search". The queries were originally published in ref. [47].
