# Supplementary material for: Speos: an ensemble graph representation learning framework to predict core gene candidates for complex diseases
Source: Nat Commun. 2023 Nov 8;14:7206. doi: 10.1038/s41467-023-42975-z (PMC10632370; doi:10.1038/s41467-023-42975-z)
Supplement: Supplementary file 30 — Reporting Summary [file 41467_2023_42975_MOESM30_ESM.pdf]

nature portfolio

Corresponding author(s): Pascal Falter-Braun, Matthias Heinig

Last updated by author(s): Oct 6, 2023

Reporting Summary

Nature Portfolio wishes to improve the reproducibility of the work that we publish. This form provides structure for consistency and transparency in reporting. For further information on Nature Portfolio policies, see our Editorial Policies and the Editorial Policy Checklist.

Statistics

For all statistical analyses, confirm that the following items are present in the figure legend, table legend, main text, or Methods section.

n/a	Confirmed	
☐	☒	The exact sample size (*n*) for each experimental group/condition, given as a discrete number and unit of measurement
☒	☐	A statement on whether measurements were taken from distinct samples or whether the same sample was measured repeatedly
☐	☒	The statistical test(s) used AND whether they are one- or two-sided<br>*Only common tests should be described solely by name; describe more complex techniques in the Methods section.*
☒	☐	A description of all covariates tested
☐	☒	A description of any assumptions or corrections, such as tests of normality and adjustment for multiple comparisons
☐	☒	A full description of the statistical parameters including central tendency (e.g. means) or other basic estimates (e.g. regression coefficient) AND variation (e.g. standard deviation) or associated estimates of uncertainty (e.g. confidence intervals)
☐	☒	For null hypothesis testing, the test statistic (e.g. *F*, *t*, *r*) with confidence intervals, effect sizes, degrees of freedom and *P* value noted<br>*Give P values as exact values whenever suitable.*
☒	☐	For Bayesian analysis, information on the choice of priors and Markov chain Monte Carlo settings
☒	☐	For hierarchical and complex designs, identification of the appropriate level for tests and full reporting of outcomes
☒	☐	Estimates of effect sizes (e.g. Cohen's *d*, Pearson's *r*), indicating how they were calculated

Our web collection on statistics for biologists contains articles on many of the points above.

Software and code

Policy information about availability of computer code

Data collection	We gathered the datasets manually from the sources described in the Data Availability Statement
Data analysis	The analysis has been conducted with Speos V0.9.2 which is available on Github (https://github.com/fratajcz/speos/releases/tag/v0.9.2) and zenodo (https://doi.org/10.5281/zenodo.8416439)

For manuscripts utilizing custom algorithms or software that are central to the research but not yet described in published literature, software must be made available to editors and reviewers. We strongly encourage code deposition in a community repository (e.g. GitHub). See the Nature Portfolio guidelines for submitting code & software for further information.

Data

Policy information about availability of data

All manuscripts must include a data availability statement. This statement should provide the following information, where applicable:

- Accession codes, unique identifiers, or web links for publicly available datasets
- A description of any restrictions on data availability
- For clinical datasets or third party data, please ensure that the statement adheres to our policy

All datasets used in this study are already published and were obtained from public data repositories. Edges and Networks: BioPlex 3.0 edgelists are available at https://bioplex.hms.harvard.edu/interactions.php . HuRI edgelist is available at http://www.interactome-atlas.org/download . Intact edgelist is available at ftp://ftp.ebi.ac.uk/pub/databases/intact/current/psimitab/intact.txt . GRNdb edgelists are available at http://grndb.com/download/ . Hetionet edgelist is available at

https://github.com/hetio/hetionet/tree/master/hetnet/tsv . Nodes and Features: Full list of human protein-coding genes is available at https://www.genenames.org/download/statistics-and-files/ , accessed 18.3.22. Positive labels are available at https://github.com/bogdanlab/gene_sets/tree/master/mendelian_gene_sets , accessed 17.3.22. GWAS summary statistics are available at https://doi.org/10.5281/zenodo.3629742 . Tissue-specific median gene expression values are available at https://storage.googleapis.com/gtex_analysis_v7/rna_seq_data/GTEx_Analysis_2016-01-15_v7_RNASeQCv1.1.8_gene_median_tpm.gct.gz . Median gene expression in blood cells is available at https://v19.proteinatlas.org/download/rna_blood_cell.tsv.zip , accessed 17.3.22. External validation: Mouse knockout genes are available at http://www.informatics.jax.org/allele , accessed 17.3.22. Lists of differentially expressed genes were downloaded from https://gemma.msl.ubc.ca/phenotypes.html , accessed 2.8.22. LoF and Missense Mutation intolerance Z-scores are available at ftp://ftp.broadinstitute.org/pub/ExAC_release/release1/manuscript_data/forweb_cleaned_exac_r03_march16_z_data_pLI.txt.gz . List of drug targets are available at https://dgl-data.s3-us-west-2.amazonaws.com/dataset/DRKG/drkg.tar.gz . Lists of druggable genes are available at https://www.dgidb.org/downloads , accessed 24.3.22.

For reproducibility, the data can be jointly obtained via Speos' repository: https://github.com/fratajcz/speos or in its processed form from https://doi.org/10.5281/zenodo.7468127 .

Research involving human participants, their data, or biological material

Policy information about studies with human participants or human data. See also policy information about sex, gender (identity/presentation), and sexual orientation and race, ethnicity and racism.

Reporting on sex and gender	No data related to sex or gender was evaluated in this study
Reporting on race, ethnicity, or other socially relevant groupings	No human research participants were recruited specifically for this study. While GWAS data used in this study is population-based, this is not identical to race and/or ethnicity.
Population characteristics	No human research participants were recruited specifically for this study. Population based results were obtained from published GWAS summary statistics.
Recruitment	No human research participants were recruited specifically for this study.
Ethics oversight	No human research participants were recruited specifically for this study.

Note that full information on the approval of the study protocol must also be provided in the manuscript.

Field-specific reporting

Please select the one below that is the best fit for your research. If you are not sure, read the appropriate sections before making your selection.

☒ Life sciences ☐ Behavioural & social sciences ☐ Ecological, evolutionary & environmental sciences

For a reference copy of the document with all sections, see nature.com/documents/nr-reporting-summary-flat.pdf

Life sciences study design

All studies must disclose on these points even when the disclosure is negative.

Sample size	This study describes analyses on human protein coding genes. No sample size calculations were performed, but we selected data sources which have the largest sample size available for the type of data they contain (i.e. GTEx for gene expression, selected GWAS studies etc).
Data exclusions	Genes were excluded from the study if any relevant input feature (GWAS, gene expression) was missing.
Replication	The experiments were replicated with each of the six methods for each of the 5 (7 including Supplementary Material) disease groups. Validation was performed against independent datasets. Replication of the results using the same settings was not performed.
Randomization	We did not handle samples or individual participant data, therefore, randomizing such data is not possible. All data is aggregated to population level.
Blinding	We did not handle samples or individual participants, therefore, blinding of participants, samples or interviewers is not possible.

Reporting for specific materials, systems and methods

We require information from authors about some types of materials, experimental systems and methods used in many studies. Here, indicate whether each material, system or method listed is relevant to your study. If you are not sure if a list item applies to your research, read the appropriate section before selecting a response.

Materials & experimental systems

n/a	Involved in the study
☒	☐ Antibodies
☒	☐ Eukaryotic cell lines
☒	☐ Palaeontology and archaeology
☒	☐ Animals and other organisms
☒	☐ Clinical data
☒	☐ Dual use research of concern
☒	☐ Plants

Methods

n/a	Involved in the study
☒	☐ ChIP-seq
☒	☐ Flow cytometry
☒	☐ MRI-based neuroimaging

Plants

Seed stocks

This study does not involve plants.

Novel plant genotypes

This study does not involve plants.

Authentication

This study does not involve plants.
